# Machine learning models for predicting the hardness of additively manufactured Polymethyl Methacrylate (PMMA) parts for biomedical applications

**DOI:** 10.1371/journal.pone.0355862

**Published:** 2026-08-13

**Authors:** Dhinakaran Veeman, Pradeep Castro Ponnuswamy, Murugan Vellaisamy, Shivashiga Aravind Kumar Mangayarkarasi, Jitendra Kumar Katiyar

**Affiliations:** 1 Centre for Additive Manufacturing, Chennai Institute of Technology, Chennai, India; 2 Manipal Institute of Technology, Manipal Academy of Higher Education, Manipal, India; Instituto Federal do Espírito Santo: Instituto Federal de Educacao Ciencia e Tecnologia do Espirito Santo, BRAZIL

## Abstract

Additively fabricated Polymethyl Methacrylate (PMMA) is highly preferred for medical applications. Its biocompatibility makes it the preferred material for biomedical applications such as prosthetics and dentistry. It is crucial to understand the mechanical properties of such materials when used in biomedical applications. Determining the mechanical properties of parts is a time-consuming and costly process. Additive manufacturing involves various process parameters that directly influence the mechanical properties of the fabricated parts. Hence, relying on traditional methods to determine the mechanical properties of additively fabricated parts would be ineffective. With advancements in technology, optimizing the process parameters for additive manufacturing of components would simplify the manufacturing process. Similarly, to determine the mechanical properties of materials, technological advancements must be integrated with additive manufacturing. Although non-destructive tests exist, they have limited applications and are too costly. This investigation analyses the feasibility of using machine learning models to predict the hardness of additively fabricated polymethyl methacrylate. Layer height, infill density, infill pattern, and raster orientation or infill line direction strongly influence mechanical properties and are considered variable input process parameters. The influence of each parameter on the hardness of the material is described. The machine learning models are evaluated using metrics to determine the best-fit model. This investigation would help medical experts determine the most suitable process parameters for additively fabricating Polymethyl Methacrylate parts for biomedical applications. Additionally, this investigation would help experts vary the hardness of biomedical parts according to the requirements or specific applications.

## Introduction

Additive Manufacturing (AM) is an advanced manufacturing process in which the products are fabricated layer-by-layer. This method differs drastically from conventional subtractive manufacturing, in which the product is fabricated by removing unwanted material from the raw material. AM is frequently used to create complicated and sophisticated structures that are challenging to create with subtractive manufacturing. The fundamental benefit of this approach is that it causes less material wastage than conventional subtractive manufacturing. There are seven types of AM techniques: Photopolymerization, Sheet lamination, Powder Bed Fusion (PBF), Directed Energy Deposition (DED), Material Jetting, Binder Jetting and Material Extrusion [1]. The prominent additive manufacturing technique called Fused Filament Fabrication (FFF) falls under the Material Extrusion category. It builds objects layer by layer by fusing and depositing material. The main raw materials used in this process are thermoplastic polymers, which are in the form of filaments. Thermoplastic polyurethane (TPU), Thermoplastic Elastomers (TPE), Polylactic Acid (PLA), Polymethyl Methacrylate (PMMA), Polyethylene Terephthalate Glycol (PETG), and Acrylonitrile Butadiene Styrene (ABS) are examples of common polymers utilized. The required CAD model is designed using any design software and then imported into a slicing software. The software divides the part into several layers by defined process parameters to create a route for the machine to follow. The machine parts move through the predefined path and create the required model. This process enables the production of custom-made goods with intricate geometries for a variety of uses in the manufacturing, medical, and prototyping sectors. The defined process parameters, like layer height, print speed, etc., have a big impact on the strength of the fabricated part, its mechanical characteristics, and general quality [2]. Hence, it is important to select appropriate process parameters to attain the required quality of the part. Ashtankar et al. [3] studied the effect of build orientation on the compressive and mechanical strength of additively fabricated ABS parts. Elena et al. [4] demonstrated that to have better bending properties, a minimum layer thickness is required. They have additionally confirmed that increasing layer thickness would result in better impact properties. EsSiad et al. [5] studied the influence of layer orientation on mechanical properties by varying angles with 0°, 45°/-45°, 45°, 90°, and 45°/0° of 3D printed ABS material. Liseli et al. [6] analyzed the impact of infill patterns on the mechanical properties like tensile and compressive strength, of FDM printed parts. Wang et al. [7] have studied the influence of nozzle temperature in the FDM process. The nozzle temperature influences the part by allowing the development of internal stress, which might lead to inter- and intra-layer deformation, resulting in part failure. The raster width, as illustrated by Dey et al. [8], demonstrated that raster width in the FDM process influenced the part build time. Higher values of raster width reduced the part build time. Most researchers have used the Taguchi method and ANOVA procedures to optimize the process parameters in the FDM process. However, optimizing FDM process parameters to improve the quality of parts was costly and time-consuming. Thus, researchers have diverted their attention towards Artificial Intelligence for process parameter modelling and optimization. Batu et al. [9] have recommended that Artificial Intelligence (AI) and machine learning (ML) would offer accurate and effective results in the optimization of FDM process parameters by detecting complex behaviour in data, making them an ideal tool for manufacturers.

Machine learning is a significant element of artificial intelligence, which functions primarily based on pattern generalization. Various datasets are provided to machine learning algorithms, which afterwards examine the datasets to discover and yield generalized patterns. The model learns these patterns as the foundation for handling and processing newly discovered data. The machine learning algorithm can use its discovered patterns to make educated judgments or predictions about fresh inputs by applying the insights from the original data [10]. The algorithm learns from its past practices and improves its algorithmic performance. It becomes more efficient when it is exposed to more data since it becomes more proficient at identifying patterns and correlations. It will modify and enhance its performance over time because of this never-ending cycle of experience and learning. As a result, the algorithm’s outputs become more precise, producing more exact and dependable outcomes [11].

The way that machine learning algorithms are based is through the process of learning and self-improvement. They process a lot of data during the first training phase to find underlying patterns and relationships. After being trained, the algorithms can be used to produce new data and precise predictions or classifications by putting in the knowledge they have gained. Machine learning is an appreciated tool in diverse applications, from image and speech recognition to predictive analytics and autonomous systems, due to its capacity to automatically improve without explicit programming for each new case [12]. As learning is iterative, the algorithm improves with every new dataset, eventually improving its capacity to manage challenging tasks and produce accurate results.

Machine learning has several uses in manufacturing technology and greatly improves productivity, creativity and efficiency [13]. Predictive maintenance reduces downtime and increases equipment lifespan by utilizing machine learning algorithms to evaluate sensor and machinery data [14]. This allows for defect diagnosis, condition monitoring, and proactive maintenance. Machine learning aids in defect identification and process optimization in quality control, ensuring constant product quality and reducing waste. Demand forecasting, logistics, and inventory management all contribute to supply chain optimization, which lowers costs and speeds up deliveries. Robotics and adaptive control systems improve process automation and control by providing more flexibility and dynamic adjustments. Through demand forecasting, consumption efficiency, and pattern analysis, energy management is optimized. Improved Simulation models and generative design drive innovation and design by encouraging creativity and cutting down on design cycles. By forecasting interruptions and evaluating supplier performance, machine learning also helps with supply chain risk management. Virtual Reality (VR) and augmented reality (AR) applications also improve human-machine interaction by improving training and maintenance. At the same time, collaborative robots operate alongside human operators to increase productivity and safety. Manufacturing processes that use machine learning yield notable enhancements in terms of overall competitiveness, product quality, and operational [15].

Additive manufacturing enables the efficient production of complex parts with minimal time and material consumption. The material properties in 3D printing, such as brittleness, ductility, and melting temperature, are influenced by the printing method and the material’s molecular and structural characteristics. Shaping the mechanical properties of 3D-printed components comprises a comprehensive process that embraces experimental testing, material characterization, computational modelling, and quality control [16]. Experimental testing, using methods like tensile, compression, flexural, hardness, impact, fatigue, creep, fracture toughness, and non-destructive testing, is vital for assessing the mechanical properties of 3D printed materials. Nevertheless, it is often costly, time-consuming, destructive, and prone to human error [17]. Subsequently, alternative solutions such as computational modelling and simulation are desirable for a more reliable understanding of material behaviour. ML involves creating algorithms and statistical models that permit computers to learn from patterns and inferences rather than explicit programming. It meaningfully supports the prediction of the mechanical properties of 3D-printed specimens [18].

Dhinakaran et al. [19] used machine learning models such as linear regression, decision tree, random forest, and Adaboost to optimize the process parameters and to forecast the hardness of 3D printed ABS material. The results established that the random forest model had specified the most accurate predictions of the hardness value of 3D printed ABS material. Peloquin et al. [20] presented a framework that uses machine learning models for forecasting the tensile properties of 3D-printed gyroid lattices. The particulars of the base material and the structure’s porosity were utilized as the data. The predicted results displayed closeness with numerically simulated values in reduced time. Ali et al. [21] established predictive models using the Gaussian Process Regression model, Decision Tree Regression model, Support Vector Machine (SVM) model, and XGBoost Regression models for cast and printed concrete. Based on metrics such as the Coefficient of Determination (R²), Root Mean Square Error (RMSE), Mean Square Error (MSE), and Mean Absolute Error (MAE), the SVM model outperformed others. Nasrin et al. [22] used Machine learning to predict the tensile properties of additively manufactured Technomelt PA 6910. Machine learning models, including linear regression, ridge regression, Gaussian process regression, and K-nearest neighbours, were utilized to predict properties. Linear regression and ridge regression were found to be effective in the prediction process. Abdelhamid Ziadia et al. [23] developed ensemble learning models for predicting the ultimate tensile strength, Young’s modulus, and strain at the break of PLA and PLA-CF 3D-printed parts. Ensemble learning techniques, including XGBoost, gradient boosting regressor, random forest, decision tree, multiple linear regression, lasso, and ridge regression, were employed to achieve accurate predictions of the mechanical properties.

Several researchers have been constantly involved in utilizing machine learning models to predict material properties. Additive manufacturing has gained attention in recent years. In this research work, ML models are used to predict the hardness of additively fabricated Polymethyl Methacrylate (PMMA) parts. The predicted values of each model are related to the actual values to analyze the effectiveness of the models. The property of hardness can be very practical in relation to the field of biomedical engineering. This is especially true regarding the usage of materials such as PMMA in manufacturing dental prosthesis, bone replacement structures, and even customized biomedical parts. When considering the biomedical application, hardness becomes an extremely important attribute in determining wear resistance when working with any parts that will experience constant rubbing or friction against something else, such as dental crowns or dentures. Hardness is another factor in ensuring that the surface is durable and resistant to deformation. Regarding bone replacement structures and implants, having a certain degree of hardness ensures their compatibility. Thus, predicting hardness according to the parameters of manufacture makes it possible to optimize parts manufactured by additive manufacturing processes

### Methodology

Polymethyl methacrylate (PMMA) is selected as the candidate material for this investigation. It is a UV-resistant thermoplastic recognized for its excellent light transmission, weather resistance, and mechanical properties, as well as good tensile strength and rigidity. With a density of about 1.18 g/cm³, it is reasonably light. These physical characteristics are represented by the values of a heat capacity of 1.47 J/gK and thermal conductivity of 0.19 W/mK. However, despite the fact that the material’s brittleness is considered to be the significant negative feature, when compared to other materials, PMMA offers good chemical resistance mainly to dilute acids and alkalis. These properties make PMMA very useful in medical applications such items as lenses, dental prosthetics, and bone cements due to its biocompatibility. PMMA’s adaptability and excellent properties make it a widely applied material in numerous industries, from consumer goods to high-tech applications. The printing temperature for PMMA naturally ranges between 240–260°C, while the heated bed should be sustained at 80–100°C to confirm proper adhesion and minimize warping. A slow to moderate print speed, around 30–50 mm/s, assists in keeping layer adhesion and surface finish. In this research, the nozzle temperature was designated at 245°C while the bed temperature was kept at 90 °C. The printing speed was preserved at 40 mm/s. The properties of PMMA are ssummarized in Table 1.

**Table 1:** Properties of PMMA.

Property	Value
Density	1.18 g/cm³
Thermal Conductivity	0.19 W/mK
Specific Heat	1.47 J/gK
Print Temp	240–260°C
Bed Temp	80–100°C

**Fig 1:**
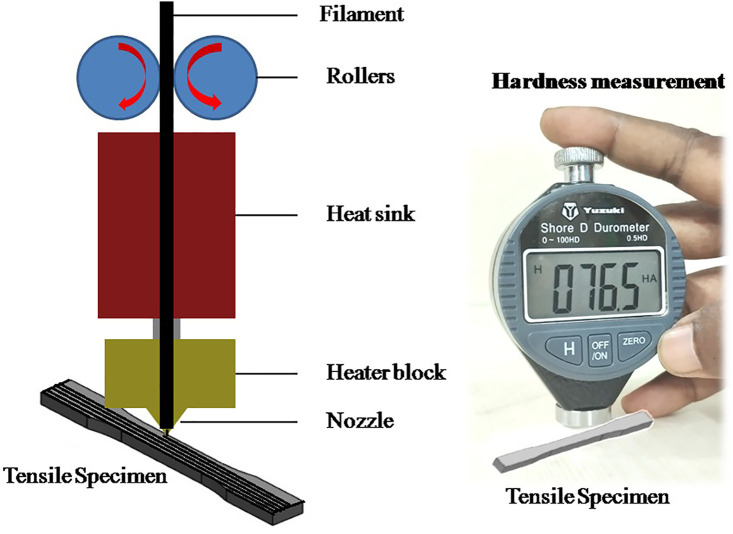
3D printing of specimen and hardness measurement using Shore D Durometer.

A Creality FDM printer with model name CR-6 SE, with a build volume of 235 x 235 x 250 mm, was used to fabricate the test specimens. The printer had a layer resolution varying from 0.1 mm to 0.4 mm with a maximum printing speed of 180 mm/s. The open chamber type FFF printer supports the use of materials such as PLA, TPU, PETG, PMMA and ABS. The variable process parameters chosen in the investigation are Layer height (0.1 mm, 0.2 mm and 0.3 mm), Infill pattern (50%, 75% and 100%), Infill pattern (Lines, Cubic, and tri-hexagonal) and Raster orientation (0°, 45°, and 90°). A total of 81 tensile specimens were printed with the variable process parameters. The hardness of each specimen was experimentally evaluated using a Yuzuki Shore D hardness meter, as shown in Fig 1.

**Fig 2:**
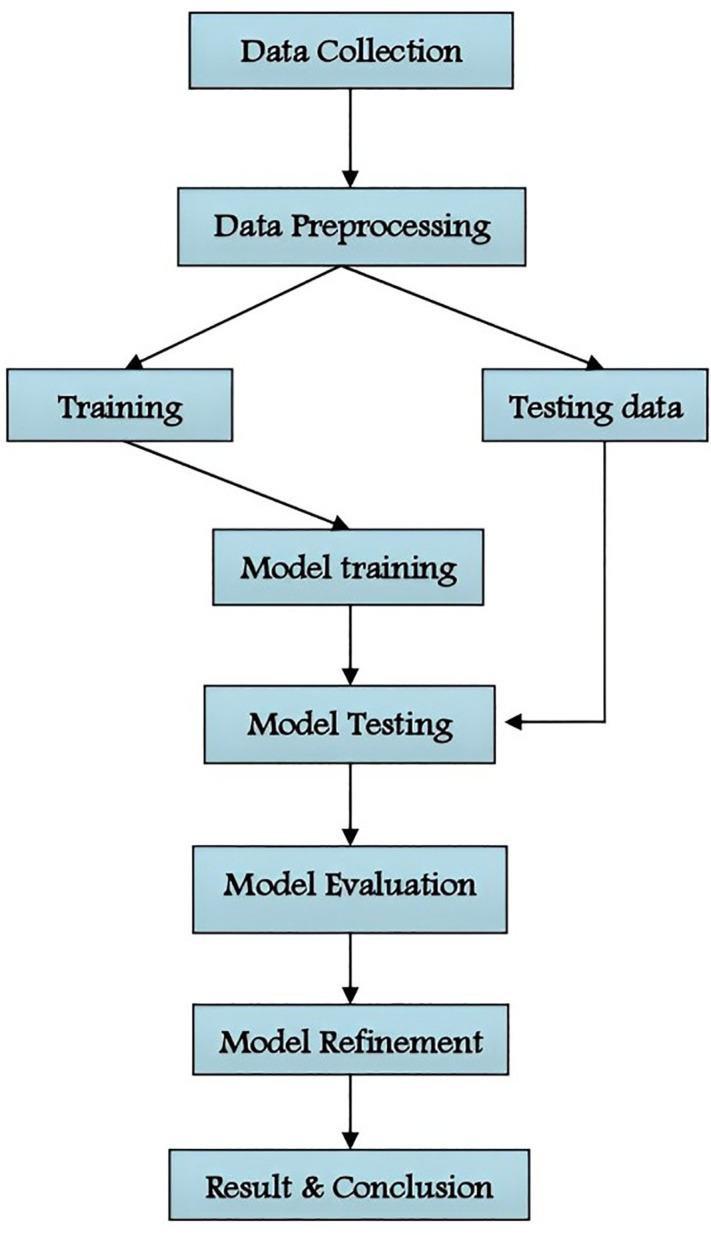
Methodology of investigation.

A predetermined force is applied to the indenter while it presses against the material on a flat, solid surface. The depth of the indentation formed by the indenter is measured both immediately after contact and after a predetermined time interval, usually 15 seconds. The durometer’s scale, which goes from 0 to 100, is then used to read the hardness value directly; greater numbers denote harder materials. The acquired experimental values were split into training and testing data for use with machine learning models. 70% of the data was used as training data and the remaining as testing data. In this research work, hardness values for the corresponding specimens were recorded thrice on different spots. This was made to ensure that any potential error due to local variations is taken into account. The values of hardness used in the analysis are the average of these three measurements. Moreover, a full factorial combination table has been included in Annexure 1 with the average value of hardness. Besides improving precision, a descriptive statistical analysis has also been done by including the mean and standard deviation. Although this research is based on predictions using machine learning models, an analysis of the parameters’ impact through statistics has also been considered. The methodology of the investigation is represented in Fig 2.

Six machine learning models were comparatively analysed in this investigation. The models were provided with training data to develop a relation between the input variables and the output initially. The models were later provided with test data to predict the output values based on the relation developed using training data. The ML models used are briefly described.

### Linear regression

In linear regression analysis, which is based on the correlation between multiple independent predictors, one target variable is employed. In a linear regression model, which is a baseline regression model, a best-fit line is produced to forecast the target variable. The number of independent factors affects how linear regression modelling is used [24].

#### Decision tree.

Decision trees address regression and classification applications. Our proposed regression model makes use of decision trees. They are more beneficial than simple regression models since they enable us to understand and interpret the logic of the algorithm. The method works by first creating branches, or conditions, from which nodes are separated to create nodes or features, and then creating leaves, or output classes or labels, from leaves. The order in which the input variables are assigned as nodes is determined using a variety of metrics [25].

### Support Vector Regression

Support Vector Machines (SVM) were the basis for the development of Support Vector Regression (SVR), an improved machine learning technique for regression applications. Unlike traditional regression methods, SVR searches for a function that deviates from actual observed targets by no more than a certain margin to guarantee a robust fit. SVR can easily handle both linear and non-linear relationships by converting the input data into higher-dimensional spaces using kernel functions such as polynomial, linear, and radial basis function (RBF) kernels. SVR’s versatility allows it to depict complex patterns in the data [26].

### AdaBoost

Each boosting technique uses a different approach to make the weak base learners stronger. Usually, the Adaboost algorithm combines its weak learners to produce one strong learner [27]. The Adaboost model performs best in regression use cases when the sample distribution is altered. Similar to random forests, Adaboost base learners are decision trees. However, each decision tree has a depth value of 1. Random forests and Adaboost differ primarily in that in the former case, each base learner is assigned an equal weight in the final output.

### XGBoost

As the name “boosting” suggests, these techniques provide a kind of augmentation for weak learners by adjusting the weights that arise from incorrect classifications so that the final result is approximately optimal. The latter is improved by combining the weighted votes for a certain categorization across iterations. Many popular and well-known boosting model types are available, including XGBoost, gradient boosting machine, light gradient boosting machine, adaptive boosting, and categorical boosting. Since XGBoost employs regularization to prevent overfitting and makes use of parallel processing to speed up performance for the target nodes, it is considered an improvement over bagging and even the boosting algorithms that were previously covered [28].

### CatBoost

Unlike standard boosting approaches that need extensive preparation of these features, CatBoost may treat categorical data directly by using an efficient encoding method based on target statistics. This approach reduces preprocessing time and maintains information, which enhances model performance. CatBoost uses ordered boosting to increase generalization and decrease target leakage [29]. Its scalability and speed enhancements, which also include GPU training and multi-threading, may allow it to handle large datasets. The algorithm employs strong regularization approaches to prevent overfitting, which results in good accuracy across a range of applications.

Though the method of k-fold cross-validation is widely used during the generalization of machine learning models, in this research, the approach was voluntarily avoided. This was due to the nature of the experimental data obtained. A full-factorial design of experiments (DOE) was followed in the current research. All process parameter combinations were considered in the experimental DOE dataset. Random partitioning used in k-fold cross-validation might violate the balance between datasets and produce sets where some combinations are not sufficiently represented. Thus, to ensure the generalization capability, a controlled train–test split in a ratio of 70:30 was employed, where all levels and parameter combinations, including layer height, infill density, infill pattern, and raster orientation, were considered in both parts. The model assessment was conducted based on the metrics of MSE and R², complemented with residual analysis to make sure there is no bias. Similarly, MSE and R² values observed in this case for both training and testing data were related to the deterministic nature of some models and balanced uniform data distribution.

## Results and discussion

**Fig 3:**
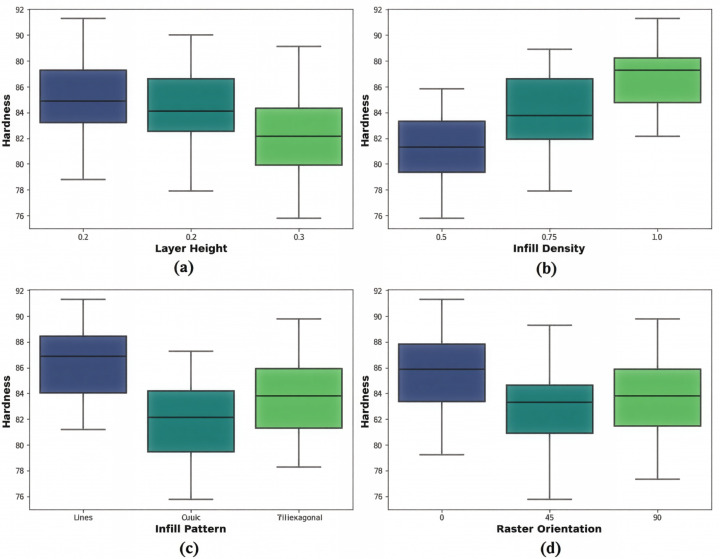
Box Plot representing the Effect of the input parameters on the hardness of PMMA material.

## Boxplot

Fig 3 represents the Box plot drawn using input parameters like Layer height, Infill Density, Infill pattern, and Raster Orientation. Fig 3(a) explains the graph drawn between the Hardness and layer height; the Impact of Various Layer Heights (0.1 mm, 0.2 mm, and 0.3 mm) on the hardness of the printed items is displayed in the boxplot for Layer Height. Below is a thorough breakdown of the hardness values for every layer height.

*0.1 mm*: For a layer height of 0.1 mm, the hardness values roughly fall between 80 and 90. This suggests that the items that are printed at this layer height have a consistently high level of toughness. With a median hardness of approximately 86, almost half of the hardness values fall below and the remaining fall over the range. The minimal variability in hardness is indicated by the narrow interquartile range (IQR), which implies that the majority of the data points are near the median.*0.2 mm*: The hardness values range around 76–90 for a layer height of 0.2 mm. Compared to 0.1 mm, this variations show a more comprehensive range of hardness values, suggesting superior variability. The median hardness is approximately 85, which is a little less than 0.1 mm. This implies that raising the layer height to 0.2 mm can raise variability while somewhat lowering overall hardness.
*0.3 mm*: The hardness values likewise vary from roughly 76–90 when the layer height is increased to 0.3 mm. Out of the three-layer heights, this one has the lowest median hardness, at about 82. This suggests that a 0.3 mm layer height typically yields higher variability and lower hardness values, as shown by the broader IQR.

From this observation of Fig 3(a), it is evident that with reduced layer height, the Hardness is higher. The layer height of 0.1 mm produces higher hardness compared to other layer heights.

Fig 3(b) explains the effect of variation in hardness with respect to modifications made in infill density.

*50% Infill:* There is a median of 82 and a range of hardness values of roughly 76–88. This shows that hardness is more varied and lower at lower infill densities.

*75% Infill:* The hardness levels have a median of 85 and range from roughly 76–90. An improvement in hardness over 50% infill density is indicated by a greater median hardness and a somewhat wider range when the infill density is increased to 75%.

*100% Infill:* There is a median of 87 and a range of approximately 80–90 for the hardness ratings. The hardness values are consistently greater and less variable at the maximum infill density, indicating that of the three densities, a full infill density yields the highest and most constant hardness.

From Fig 3(b), it is evident that the hardness of the printed object improves along with a corresponding variability when the infill density goes from 50% to 100%. This highlights how critical infill density is for producing 3D printed objects with higher hardness and more uniformity.

Fig 3(c) indicates the box plot between the infill pattern and the hardness. The box plot for the Infill Pattern parameter shows the relationship between the hardness of 3d Printed specimens among three infill patterns namely Lines, Cubic, and Tri-hexagonal. Higher hardness is found in the Lines pattern, where the median hardness is roughly 87 and ranges from about 80–90. This suggests that the Lines pattern regularly yields higher hardness than the other patterns. The Cubic pattern, on the other hand experinences moderate hardness with some variability, with hardness values ranging around 76–88, with a median of 83. The Trihexagonal pattern shows the lowest and least hardness values around 76–88 with a lower median around 82. These results imply that a higher and more consistent hardness can be achieved in 3D printed products by using the Lines infill pattern.

The box plot in Fig 3(d) illustrates how raster orientation affects the hardness of 3D printed objects. The hardness values vary from about 80–90 at 0° raster orientation, with a median value of about 87. This suggests that 3D printed components with this particular orientation typically have harder surfaces that are both more uniform and harder. In comparison to the 0° orientation, the hardness values at a 45° orientation exhibit a broader range, from 76 to 88, with a median around 83, indicating greater variability and overall lower hardness. The hardness values at 90° orientation range around 76–90, with a median of 85. Thus, a 0° raster orientation produces the highest and most consistent hardness, while a 45° orientation produces the lowest. This orientation produces slightly more hardness than the 45° orientation but still shows more variability than the 0° orientation.

### Scatter plot

**Fig 4:**
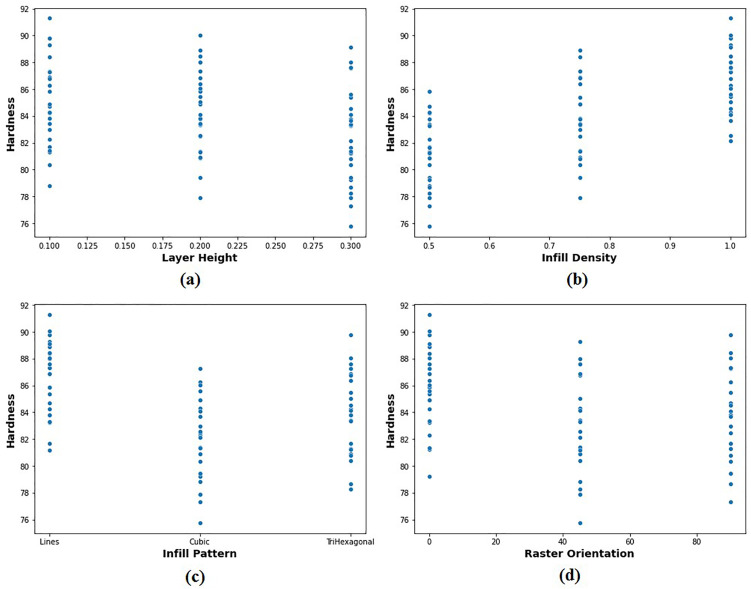
Scatter Plot representing the Effect of the input parameters on the hardness of PMMA material.

The scatter diagrams in Fig 4 provide a visual representation of the effect of four different 3D printing parameters namely layer height, infill density, infill pattern, and raster orientation on the hardness of printed objects. Each graph highlights the distribution and relationship between input parameters and output hardness values. Fig 4(a) shows the relationship between layer height (0.1 mm, 0.2 mm and 0.3 mm) and hardness values. Results indicate that objects printed at 0.1 mm layer height tend to have high hardness. Scores are closely clustered, indicating minimal variation. Hardness values of 0.2 mm show a wider distribution than 0.1 mm, indicating a greater variation in hardness. Hardness values of 0.3 mm which is similar to 0.2 mm, but with average hardness lower. These mean more variation and generally lower hardness objects that were printed at this layer height. In short, lower layer heights (0.1 mm) usually give higher and more consistent hardness values, while higher layer heights (0.3 mm) increase variability and lower hardness.

Fig 4 (b) describes the effect of different filler densities (0.5,0.75and 1.0)on the hardness values. With 50% Infill density, greater variability and generally lower hardness at lower fill densities are observed. With 75% density showing a higher median and a slightly more comprehensive range, this confirms that increasing the fill density improves hardness. At the highest fill density (100%), hardness values are consistently higher and less variable, indicating that total fill density provides the highest and most consistent hardness. Briefing, when the filling density increases from 50% to 100%, the hardness of the printed object improves, and the variation decreases accordingly. Fig 4 (c) shows the relationship between different patterns (lines, cube, trihexagonal) and hardness values. Lines pattern produces consistently higher hardness values with minimal variation. The cube pattern shows moderate hardness but some variation. The trihexagonal pattern shows the lowest and least permanent hardness of the three patterns. These results suggest that the line infill pattern provides the highest and most consistent hardness of 3D printed products. Fig 4(d) shows the variation of hardness with respect to infill orientation or raster orientation. At 0°, objects printed in this direction tend to have a higher and more uniform hardness. At 45° direction, it shows greater variation and generally lowers hardness. With 90° direction, it shows a slightly higher hardness than 45°, but still more variation compared to 0°.

From the graph, a 0° raster orientation gives the highest and most consistent hardness, while a 45° orientation gives the lowest hardness. The 90° orientation gives slightly more hardness than the 45° orientation but still shows more variation than the 0° orientation. Overall, the scatter plots give a clear picture of how each parameter affects the hardness of 3D printed objects with lower layer height, higher fill density, line fill patterns, and 0° raster direction, generally resulting in higher and more uniform hardness set of values.

### Machine learning model evaluation methods

MSE (Mean squared error) and R-squared (R^2^), also known as the coefficient of determination, are the common metrics used for evaluating machine learning models. MSE is used to measure the average of the squares of the errors, while R^2^ indicates the proportion of the variance in the dependent variable that is predictable from the independent variables. For a best fit model, the MSE should be as low as possible, indicating the lowest possible errors the machine learning model results in. For R^2^, the whole number 1 indicates the variability of the response data around its mean for a best-fit model and 0 indicates that none of the variability of the response data around its mean.

### Optuna optimizer

Hyperparameters of the machine learning algorithm can be optimized using Optuna, which is a robust hyperparameter tuning library. Through the use of the latest techniques including Bayesian optimization and TPE (Tree-Structured Parzen Estimator), Optuna swiftly identifies the optimal set of parameters, improving the efficiency of the model and saving time [30]. Being easy to use along with being compatible with popular machine learning frameworks makes it an indispensable tool for programmers to obtain maximum accuracy of their models

### Scikit-Optimize(skOpt)

An open source Python library known as Scikit-Optimize (skOpt) helps optimize hyperparameters using Bayesian Optimization. It successfully identifies the best hyperparameters for machine learning algorithms by balancing exploration and exploitation. SkOpt can work effectively along with some widely used machine learning libraries such as Scikit-Learn and contains a variety of optimizers like GP, Forest, and GBR. SkOpt plays an important role in tuning the model to its optimum performance level [31].

### MSE comparison without optimization

**Fig 5:**
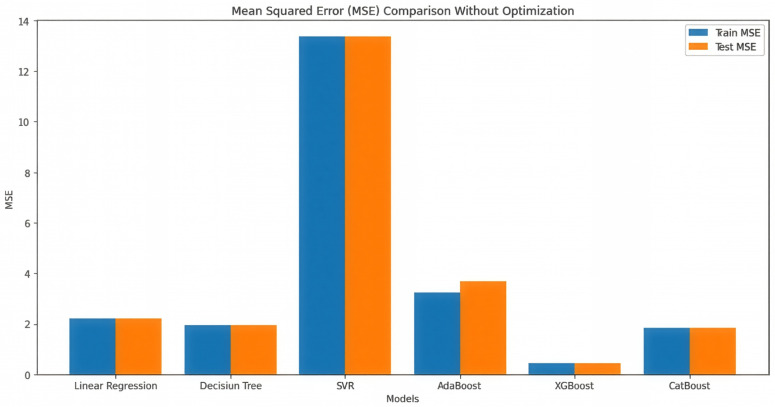
Variation of MSE for each model without optimization.

Fig 5 shows the comparison of the mean squared error (MSE) of various machine learning models before the optimization process. The various models that have been considered include linear regression, decision tree, support vector regressor (SVR), AdaBoost, XGBoost, and CatBoost. MSE is displayed separately for the training data set and test data set. According to the analysis, the linear regression and decision tree models exhibit lower and equal MSE values for the training data set and test data set, showing consistency and little overfitting. In contrast, the SVR model exhibits high MSE values for the training data set and test data set, implying poor performance and high disparity between the two sets of data.

The AdaBoost model exhibits relatively moderate MSE on training data but much larger MSE on test data, implying that the model suffers from overfitting and hence cannot perform well on unseen data. The XGBoost algorithm demonstrates quite low MSE values on the two datasets, with relatively high MSE on the test data being very impressive, implying that the model performs well and has great generalization capacity. Furthermore, CatBoost reveals low and similar MSE values on the two datasets, implying good performance and lack of overfitting. From the MSE values, it is evident that XGBoost is the best model due to its very low MSE values on both training and test datasets. Thus, despite most models performing equally well, the XGBoost algorithm dominates due to its very low MSE values.

### MSE comparison with optuna optimization

**Fig 6:**
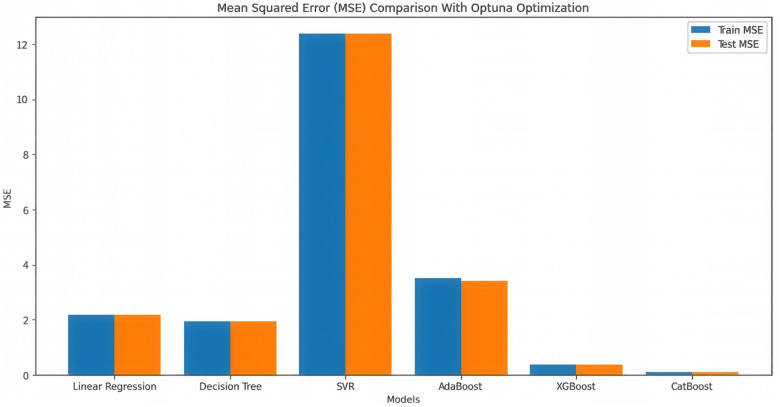
Variation of MSE with Optuna Optimization for each model.

Fig 6 illustrates a comparison of the mean squared error (MSE) for various machine learning algorithms that have been optimized using Optuna. Models under Analysis include linear regression, decision tree, support vector regression (SVR), AdaBoost, XGBoost, and CatBoost. The evaluation of the performance of each model is done in terms of both training and testing. It demonstrates how useful Optuna optimization is for all models. In linear regression, the model is inert in relation to Optuna optimization, and it produces the same results as without optimization. For the decision tree algorithm, the training MSE is close to 0, which implies overfitting without any optimization. However, with Optuna, overfitting is minimized, and the testing MSE is approximately 2.5. SVR initially shows poor generalization with a high test MSE (around 12 without optimization).

With Optuna, the training MSE and the test MSE drop to about 12, although it is still relatively high compared to the other models. This suggests that while Optuna helps, SVR still fails with generalization. AdaBoost has a base training MSE of about 3.8 and a test MSE of about 4 without optimization. Optuna significantly improves performance, training MSE tends to be the same and testing MSE reduces to 3.8. This highlights the benefit of optimization in improving model accuracy and generality. XGBoost shows an original training MSE of about 0.5 and a test MSE of about without optimization. With Optuna, the training MSE and the testing MSE remain unchanged, indicating the effortlessness of the optimization in model performance. CatBoost shows excellent results with an initial training MSE around 2 and a test MSE around 2. Optuna lowers the test MSE further, reaching the lowest value of around 0.5, indicating the best generality and performance of the evaluated models.

In General, optimization techniques like Optuna significantly improve the performance of various machine learning models. Among these models, CatBoost shows the best performance after Optuna optimization, with the lowest MSE tested, showing the most reliable generalization and the highest accuracy.

### MSE comparison with skOpt optimization

**Fig 7:**
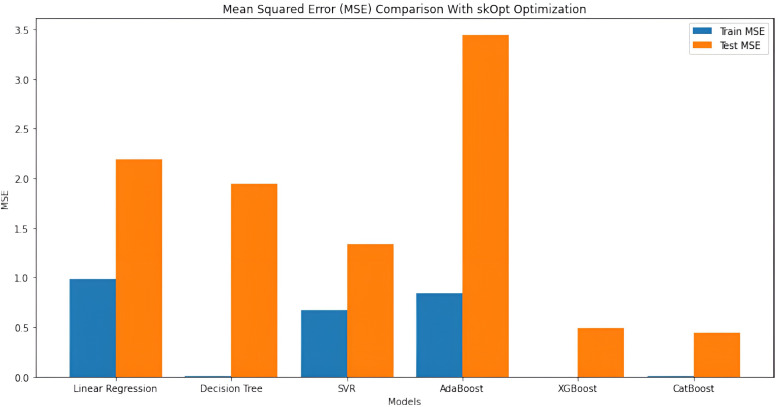
Variation of MSE with skOpt Optimization for each model.

The Fig 7 shows a comparison of the mean square error (MSE) for six different machine learning models on both training and test datasets: linear regression, decision tree, support vector regression (SVR), AdaBoost, XGBoost and CatBoost. The MSE values, which measure the root mean square difference between the actual and predicted values, are smaller for the better models. Linear regression shows potential overfitting, with a training MSE of around 1.0 and a higher test MSE of around 2.0. In contrast, it had training and testing data of 2.0, which implies that SkOpt optimization does not improve the test scores. The decision tree model has relatively high MSE values, 0.1 for training and slightly more than 2.0 for testing, which implies the optimization has improved the training score, but testing scores remain the same. SVR shows decent generalization with a low training MSE of around 0.5 and a moderate test MSE of just under 1.5. In contrast, it initially had around the value of 13, which implies SkOpt has significantly improved the performance, but it is not best compared to other models. AdaBoost shows severe overfitting, with a training MSE of just over 1.0 and a very high test MSE of around 3.5, whereas the testing score remains the same, the training score has decreased, which influences the SkOpt optimization in this model. XG Boost shines with the lowest MSE, showing almost zero training MSE and test MSE just above 0.5, indicating excellent performance generality. CatBoost also performs well, with a training MSE just below 0.5 and a test MSE just above 0.5, although slightly higher than XGBoost. Overall, XGBoost is the best model, showing the highest accuracy and generalizability, and Catboost closely follows it.

**Fig 8:**
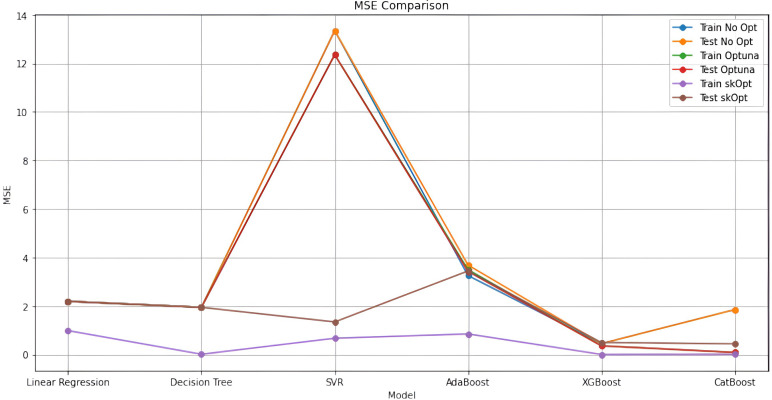
Variation of MSE for each model with and without optimizers.

Fig 8 compares the MSE of each model used for predicting the hardness of 3D printed PMMA parts. From the above observation, it can be noted that there are various optimization methods such as Optuna and skOpt which enhance the performance of various types of machine learning algorithms, with skOpt providing the best results. The linear regression model, decision tree model, AdaBoost model, XGBoost model, and CatBoost model all gain benefits from this.

### R2 without optimization

**Fig 9:**
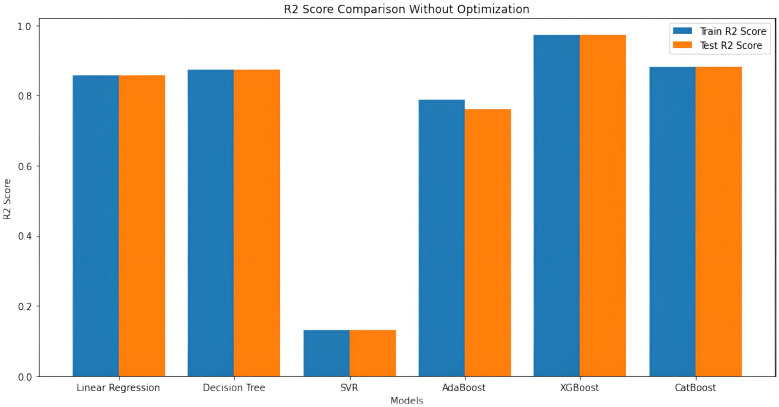
R^2^ for each model without optimization techniques.

The R² score of various machine learning models is illustrated in Fig 9 without any optimizations. The blue bars indicate the R² score of the training set whereas orange bars show the R² score of the test set. The following machine learning models have been compared in the above chart: Linear Regression, Decision Tree, Support Vector Regression (SVR), AdaBoost, XGBoost and CatBoost. According to Fig 9, it can be seen that R² score of both the training and testing are almost the same for the linear regression model which shows its consistency and efficiency. Similarly, R² score of decision tree model is quite high for both the training and testing sets showing its efficient data processing capability. On the other hand, R² scores of SVR are much less than other models which show its inefficiency. This shows that the SVR algorithm is unsuitable for such data. The R² values of the AdaBoost model are relatively good and even slightly reduced in the test set, meaning the model performance is good but not as good as those of some other models. XGBoost and CatBoost have the highest R² cores, meaning their performance is very good. They are able to produce relatively high R² values in both training and test sets, meaning they are efficient and robust despite the lack of optimization. These models are the best-performing in this case since they provide the highest R² values in both training and testing sets.

### R^2^ with Optuna optimization

**Fig 10:**
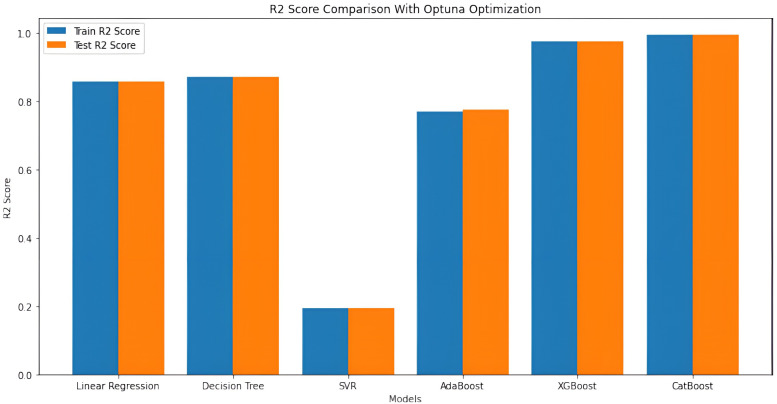
R^2^ for each model with Optuna optimization techniques.

Fig 10 depicts the comparison of the R2 score of various machine learning algorithms after optimization by Optuna. Models Compared consist of Linear Regression, Decision Tree, Support Vector Regression (SVR), AdaBoost, XGBoost, and CatBoost. It can be seen that the R2 scores for training and testing are almost similar, implying that there is uniform performance in all the tests. This uniform performance implies that there is no overfitting of the model and that it is performing well on training and testing both. Likewise, the decision tree algorithm also has almost similar high R² scores for both training and testing, suggesting that there is no effect of Optuna optimization in this algorithm. This shows that the model can capture the underlying patterns in the data without overfitting. In contrast, Even though the Optuna optimization had increased performance, the SVR model has significantly lower R² scores than the other models, indicating poor performance. This suggests that the SVR model does not fit the given data set or problem well. On the other hand, the AdaBoost model has a moderate R² score indicating decent performance with training and testing scores of nearly 0.8 and 0.9, which is high compared to the score without optimization. Although not as high as some other models, it still works pretty well. XGBoost and CatBoost, in particular, show the highest R² scores, indicating excellent performance with excellent generality. These models achieve high R² scores on both the training and test datasets, compared to the previous scores without optimization, demonstrating their efficiency and robustness after Optuna optimization. Among these models, XGBoost and CatBoost are the best performers, as they achieve the highest R² scores in both the training and test datasets, demonstrating their ability to generalize well to unseen data by capturing complex patterns in training data.

### R^2^ with SkOpt optimization

**Fig 11:**
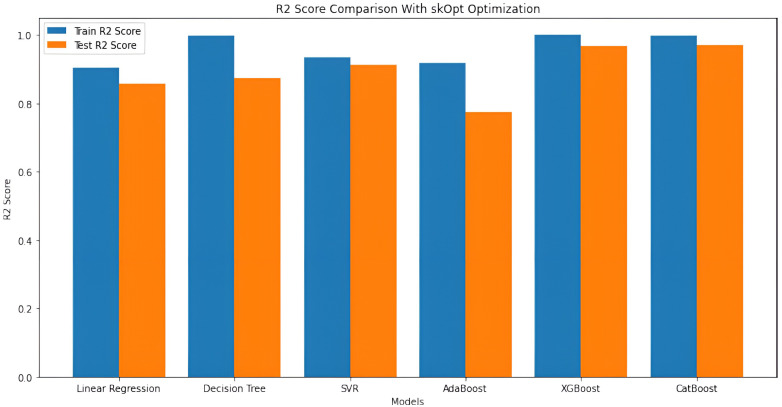
R^2^ for each model with skOpt optimization techniques.

Fig 11 shows the comparison of the R² score in different machine learning models after skOpt optimization. From The graph, we can observe that linear regression after SkOpt optimization has increased training scores, indicating the impact of the SkOpt optimization. The decision tree model also shows a high training score after SkOpt optimization, after optimization. The SVR model has shown drastically high performance compared to the scores after optimization, and SkOpt optimization has effectively changed the performance of the model. The AdaBoost model shows higher training scores compared to the model without optimization, therefore improving the performance of the model. Both XGBoost and CatBoost show high R² scores in the training set, but there is a slight decrease in the test set, indicating strong performance and good generalization ability. Among these models, XGBoost and CatBoost are the best performers after skOpt optimization, achieving high R² scores in both the training and test datasets. This indicates that they are effective at capturing underlying data patterns and generalizing to new, previously unseen data.

**Fig 12:**
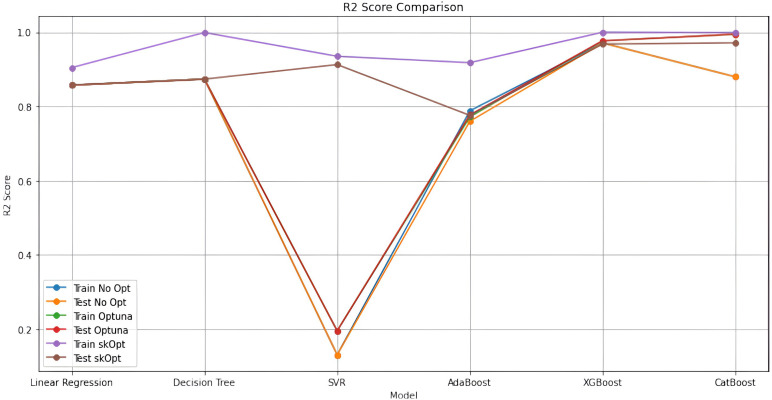
R^2^ for each model with and without optimization techniques.

Fig 12 shows the comparison of R² scores of various machine learning models with regard to three different scenarios – no optimization, Optuna optimization, and Scikit-learn optimization. On the basis of the R² scores, it can be said that the XGBoost and CatBoost models are the best-performing models. This is because both these models show high and consistent R² scores for both the training set and the test set in all three optimization scenarios, which means that both models are good at predicting and generalize well to the unseen data, respectively. However, they suffer from little overfitting and thus can gain slightly from optimization. Better results attained by CatBoost relative to XGBoost following hyperparameter optimization are based on the efficiency that the algorithm has in handling categorical features using ordered encoding. For instance, the infill pattern is handled using ordered encoding. Further, CatBoost utilizes ordered boosting, thereby minimizing chances of overfitting and ensuring good generalization, especially in small and structured datasets like the full-factorial data employed in the study. Symmetry in tree construction, as well as inherent regularization, increases stability. This makes it possible for CatBoost to produce better results, especially when optimizing algorithms such as Optuna and SkOpt are used

The models evaluated and their metric scores are listed in Table 2. From the table, it is evident that the XGBoost model with MSE = 0.4447 and R2 = 0.9710 is the best-fit model when no optimizers are used. However, when Optuna optimizer was used, CatBoost outperformed XGBoost in becoming the best model, with MSE = 0.0816 and R^2^ = 0.9947. Similarly, with the skOpt optimizer, CatBoost has responded well to become the best-fit model with MSE = 0.4397 and R^2^ = 0.9713.

**Table 2:** Comparison of evaluated metrics for each model.

Machine Learning model	MSE Values						R2 Values					
	Without Optimizer		With Optuna Optimizer		With skOpt Optimizer		Without Optimizer		With Optuna Optimizer		With skOpt Optimizer	
	Train	Test	Train	Test	Train	Test	Train	Test	Train	Test	Train	Test
**Linear Regression**	2.1884	2.1884	2.1884	2.1884	0.9815	2.1884	0.8574	0.8574	0.8574	0.8574	0.9046	0.8574
**Descision Tree**	1.9414	1.9414	1.9414	1.9414	0.0079	1.9414	0.8735	0.8735	0.8735	0.8735	0.9992	0.8735
**Support Vector Regression (SVR)**	13.3508	13.3508	12.3584	12.3584	0.6676	1.3397	0.1300	0.1300	0.1947	0.1947	0.9351	0.9127
**AdaBoost**	3.2511	3.6852	3.4975	3.4203	0.8448	3.4407	0.7881	0.7599	0.7721	0.7771	0.9179	0.7758
**XGBoost**	0.4447	**0.4447**	0.3555	0.3555	0.0000	0.4891	0.9710	**0.9710**	0.9768	0.9768	1.0000	0.9681
**CatBoost**	1.8454	1.8454	0.0816	**0.0816**	0.0053	**0.4397**	0.8797	0.8797	0.9947	**0.9947**	0.9995	**0.9713**

### Residuals comparison for all models

**Fig 13:**
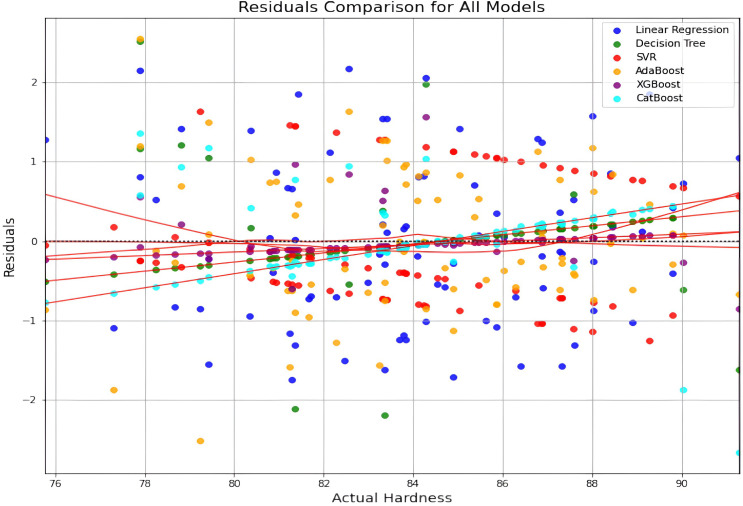
Residual comparison for all models without optimizer.

Fig 13 compares the residuals of different machine-learning models with the actual hardness values. Residual values are the differences between actual and predicted values. A good model has residuals close to zero, indicating accurate predictions. Based on the plot, CatBoost and XGBoost show residuals that are closest to zero and are relatively evenly distributed, suggesting that they are the most accurate models for predicting hardness in this data set.

They have little width and no distinctive patterns, which suggests a good fit. SVR also performs well but has slightly higher residuals. In contrast, linear regression and decision trees show higher scatter and lower accuracy. Linear Regression, despite being simple, does not necessarily account for the intricacy of the data and therefore has a bigger variance and bigger residuals. Decision Tree models suffer from the problem of overfitting due to scatterings and big residuals. AdaBoost exhibits the greatest spread and pattern and thus can be considered the least efficient model out of the three models under consideration here. The sequential learning of AdaBoost increases the chances of making mistakes and thus makes the prediction less stable. Therefore, CatBoost and XGBoost can be called the most efficient models as they have no residuals and do not have any bias/variance pattern.

### Residual comparison of all models with Optuna Optimization

**Fig 14:**
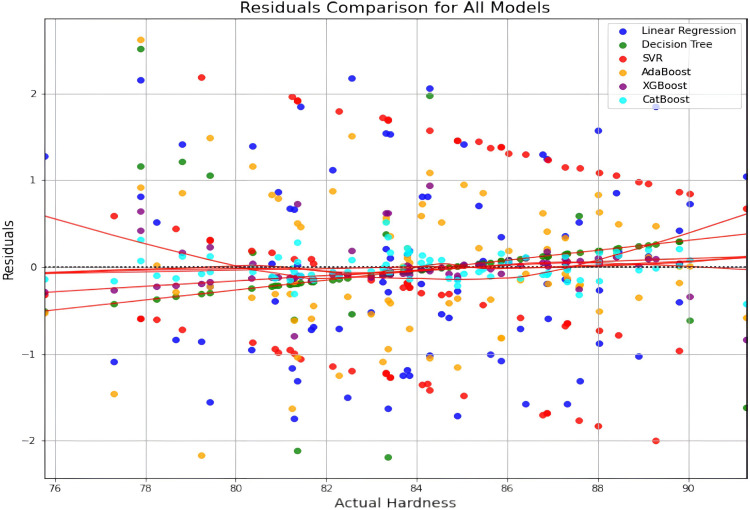
Residual comparison for all models with optuna optimizer.

The comparison of the residual values of various models with the hardness value is shown in Fig 14 where Optuna was used to optimize the hyperparameters of the algorithm. The residual value is defined as the difference between the actual value and the predicted one and helps determine the accuracy of the model. It can be seen that the most accurate models are CatBoost and XGBoost since their residual values tend to be close to zero and evenly distributed. They efficiently manage the complexity of the data, and thus provide the best prediction results. SVM also works well but with somewhat higher residual values than CatBoost and XGBoost. Linear Regression has moderate accuracy with higher variance. The decision tree shows a wider distribution of residuals, indicating overfitting and higher variance. AdaBoost performs the worst, with a noticeable residual pattern and the largest spread, suggesting that it inflates the error and leads to unstable predictions. Therefore, CatBoost and XGBoost are the most reliable models for hardness prediction when SVR is a secondary choice. At the same time, linear regression, decision tree and AdaBoost are less suitable due to higher residuals and variance.

Residual comparison of all models with SkOpt optimization

**Fig 15:**
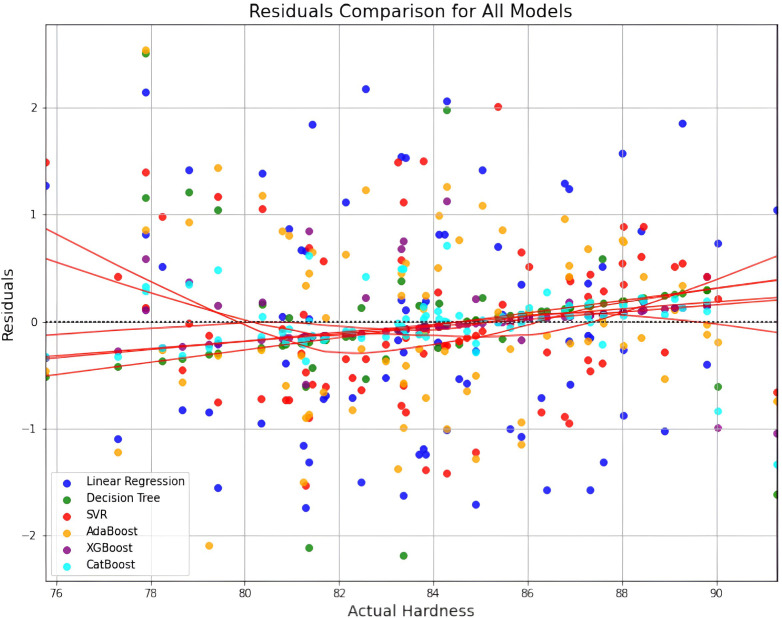
Residual comparison for all models with skOpt Optimizer.

Fig 15 illustrates a comparison of the residuals of different predictive models of “true hardness,” where the residuals show differences between the actual model values and the predicted values. The zero points scattered around the horizontal line show how accurate each model’s predictions are, and the remaining lines highlight each model’s trend. When evaluating a plot, the best model is usually identified by having the residuals most evenly distributed around zero, with no clear pattern and the smallest residual values. Linear regression and decision trees show a broader and more uneven distribution of residuals. In contrast, SVR, XGBoost, and CatBoost have more concentrated and smoother residuals of around zero. In Particular, CatBoost shows the most evenly distributed residual around zero, suggesting that it may be one of the best models for predicting true hardness.

## ANOVA analysis

Analysis of Variance, or ANOVA is an extensively used statistical tool that helps to identify the difference in the response variable caused significantly by one or more factors of the experiment. It accomplishes this by comparing the effect of variations produced by process parameters with the variance produced by errors in the experiment. This will provide the ability to identify the factors that affect the response variable in a statistically significant manner. ANOVA is extensively used in manufacturing and materials engineering research for studying the effect of process parameters.

Additive manufacturing (AM) is influenced by a number of parameters that might affect the output. Hence, it is important to analyze the important process parameters in AM, for which ANOVA plays a major role. The basic concept of ANOVA is to divide the total variation within the experimental observations in parts according to their origin. The statistical significance of factors is assessed based on the *F* statistic, which is defined as the ratio of variation attributed to a factor over the residual variance. Larger values of *F* show higher impact of the factor on the response variable. The significance of factors is then evaluated based on the associated p-value, which is typically set at 95% (α = 0.05).

In this work, ANOVA has been employed for assessing the effect of the four parameters, Layer Height, Infill Density, Infill Pattern, and Raster Orientation, on the hardness value of melt-extruded PMMA test samples. The experimental design included 81 experiments in total and was applied to study the effect of the parameters as well as their interactions. The ANOVA analysis was conducted using the Python programming language with the assistance of two libraries, namely Statsmodels and SciPy, at a 95% confidence level. The outcomes are presented in Table 3.

**Table 3:** ANOVA results.

Source	Sum of Squares (SS)	Degrees of Freedom (DF)	Mean Square(MS)	F-value	η²	p-value
Layer Height	116.553	2	58.277	149.658	0.125	<0.001
Infill Density	388.984	2	194.492	499.471	0.419	<0.001
Infill Pattern	282.336	2	141.168	362.530	0.304	<0.001
Raster Orientation	113.259	2	56.629	145.429	0.122	<0.001
Error	28.037	72	0.389	–		–
Total	929.169	80	–	–		–

### Detailed discussion of ANOVA results

It can be observed from the ANOVA analysis that all the parameters of the FDM process used in the study have a statistically significant effect on the hardness of PMMA samples. Of the various parameters analyzed, Infill Density has the highest impact on hardness variability with η² = 0.419, indicating that 41.9% of the total variation of hardness could be explained by changes in the density of infill. Other parameters with a stronger influence include Infill Pattern with η² = 0.304, Layer Height with η² = 0.125 and Raster Orientation with η² = 0.122. The large influence of infill density clearly indicates that the material distribution within the specimen, avoiding voids, has a great impact on hardness values. Additionally, the effect of infill pattern proves that filament arrangement and loading conditions have a strong effect on the hardness of FFF-printed parts. The layer height and raster orientation have a notable effect, since they greatly influence bonding between layers and filament orientation. The residual error is found to be low at only 3.02% of the total variance, which proves that the experiment conducted has high consistency. Thus, the selected process parameters explain all variations of hardness. The results of the analysis of variance show that nearly 97% of the variance in hardness could be explained by the analyzed process parameters.

### Influence of Layer Height on Hardness

The impact of layer height on hardness is depicted in Table 4. Mean hardness values found at the layer heights of 0.1 mm, 0.2 mm, and 0.3 mm were 85.24, 84.44, and 82.39 Shore D, respectively. From Table X4, it can be observed that an increase in layer height leads to a decrease in hardness. Maximum hardness is observed when the layer height equals 0.1 mm, while minimum hardness is reported at 0.3 mm. As compared to the 0.3 mm layer height, the 0.1 mm layer height allows improving hardness of about 3.46%. The 95% confidence intervals allow proving the reliability of experimental results and the low variability within each factor level. Improved hardness at lower layer heights is due to better interlayer fusion, less porosity, and more effective consolidation of the material due to smaller deposited layers. An increase in layer height causes a reduction in the bonding area between adjacent layers, which leads to poor interfacial adhesion and less resistance to indentation. According to the results of ANOVA, layer height has a significant effect on hardness with F = 149.658, p < 0.001, η² = 0.125. Thus, it is clear that lowering layer height provides enhanced hardness of additively manufactured PMMA parts

**Table 4:** Impact of layer height on hardness.

Level	Mean Hardness	95% CI
0.1 mm	85.24	84.00–86.48
0.2 mm	84.44	83.23–85.64
0.3 mm	82.39	81.01–83.77

### Infill density

From Table 5, the average hardness values determined for the infill density equaling 50%, 75%, and 100% are 81.38, 83.95, and 86.74 Shore D, respectively. The results show that hardness significantly increases as the infill density increases. The maximal hardness was achieved when the infill density was 100%, while the minimal hardness was measured at the infill density of 50%. As compared with 50% infill, the hardness increases by about 6.59% when the infill is 100%. The increase in hardness with an increase in the infill density is related to better consolidation of the material and its lower porosity. The increase in the infill density leads to a denser structure of the print. Based on the output of the ANOVA test, it is clear that infill density is the most influential process parameter, F = 499.471, p < 0.001 and has an effect size of η² = 0.419. This indicates that about 41.9% of the total variability in hardness is determined by the changes in the infill density. Hence, infill density has a major impact on the hardness of FDM-printed PMMA parts.

**Table 5:** Impact of Infill density on hardness.

Level	Mean	95% CI
50%	81.38	80.34–82.42
75%	83.95	82.83–85.07
100%	86.74	85.79–87.70

### Infill pattern

The effect of the infill pattern on the hardness parameter is shown in Table 6. The mean values of hardness achieved in the cases of the Lines, Tri-Hexagonal, and Cubic patterns were 86.43, 83.77, and 81.87 Shore D, respectively. The Lines pattern has the highest value of hardness, while the Cubic pattern has the lowest one. The Lines pattern showed approximately a 5.57% increase in hardness when compared with the Cubic pattern. The higher hardness in the Lines pattern is due to the continuous fiber structure, which facilitates load transfer. On the contrary, the Cubic and Tri-Hexagonal patterns had more complicated internal structures, leading to the formation of more interfaces and regions of local stress concentration. ANOVA analysis proves that there was a significant difference among the patterns in terms of hardness with F = 362.530, p < 0.001 and effect size of η² = 0.304. This confirms that approximately 30.4% of the total variance in hardness is attributed to the use of the selected infill pattern. It can be concluded that the internal structure geometry has an important effect on the hardness of FDM parts.

**Table 6:** Impact of Infill pattern on hardness.

Pattern	Mean Hardness	95% CI
Lines	86.43	85.39–87.47
Tri-Hexagonal	83.77	82.57–84.97
Cubic	81.87	80.66–83.08

### Raster orientation

The effect of the orientation of raster on the hardness of the composite is provided in Table 7. The average values of hardness that were measured for raster orientation of 0°, 45°, and 90° were 85.63, 82.81, and 83.64 Shore D, respectively. The maximum value of hardness was attained when 0° raster orientation was used, whereas the minimum value of hardness was obtained when 45° raster orientation was used. Compared to 45° raster orientation, the use of 0° orientation has increased hardness of about 3.41%. Better hardness values at 0° orientation can be due to the filaments’ continuity and load transmission underneath the indented area. ANOVA findings showed that raster orientation had a significant impact on hardness with F = 145.429, p < 0.001 and effect size being η² = 0.122. This means that about 12.2% of the overall variation in hardness can be accounted for by raster orientation. Even though raster orientation does not have much impact compared to infill density and pattern, it still plays an important role in determining the hardness of PMMA components fabricated via FDM.

**Table 7:** Impact of Raster orientation on hardness.

Orientation	Mean Hardness	95% Confidence Interval
0°	85.63	84.42–86.83
90°	83.64	82.37–84.90
45°	82.81	81.45–84.17

### Interaction effect analysis

To determine the joint influence of process parameters on hardness, an analysis of interaction was carried out between Layer Height and Infill Density, as the two parameters were the most influential for higher hardness. It is established from Table 8 that the interaction term was highly significant with F = 24.57, p < 0.0001. As a result, the influence of layer height on hardness is determined by a certain level of infill density, too. Thus, the two factors are not independent. The interaction effect is explained by the fact that the two processes occur simultaneously: inter-layer bonding and internal material consolidation. Smaller layer heights ensure better filament bonding and less void formation, while higher infill densities ensure better material consolidation and less porosity. Thus, the positive effect on hardness provided by smaller layer heights becomes more prominent with increasing infill density. The resulting synergy leads to interaction. The interaction effect explains about 1.78% of the variability in hardness. This value is smaller than the contributions of main effects, but due to the high F-value and extremely small p-value, the interaction effect of Layer Height and Infill Density is statistically significant. The above results show that process parameters should be optimized together rather than individually to maximize hardness in FDM-fabricated PMMA parts.

**Table 8:** Interaction effect analysis.

Source	DF	F-value	p-value
Layer Height × Infill Density	4	24.57	<0.0001

## Cross-validation for model generalization assessment

As an additional measure to validate the reliability and generalization abilities of the developed machine learning models, 10-fold cross-validation was performed alongside the train/test split method of evaluating the models’ performance. The use of cross-validation is especially important when a small experimental dataset is used, as it eliminates any possible bias related to the train/test split division and provides better results. When performing 10-fold cross-validation, the entire set of 81 experimental measurements was randomly split into ten roughly equal folds. In each round of the cross-validation process, nine out of ten folds, which is 90% of the data set were taken for training the model, whereas the other 10% was used for testing the performance of the model. This process was repeated ten times so that each fold was used for model validation once. The use of 10-fold cross-validation has several benefits when compared to a single train-test division. This approach guarantees that all observations in the experiment are used for training and validating the model. Additionally, it avoids the impact of random data splits, allows a more stable assessment of the predictive accuracy, and helps to recognize possible overfitting. Therefore, the obtained scores by cross-validation can be regarded as a better prediction of the models’ generalization on the small experimental data set. The predictive accuracy of each model was estimated through MSE and R² values

### Comparative performance of models after cross-validation

#### Linear regression – before and after 10-fold cross-validation.

Table 9 presents the performance of the Linear Regression model both prior to and after the application of 10-fold cross-validation based on the three optimization cases: unoptimized, Optuna optimized, and skopt optimized. Without the use of cross-validation, there was no difference between the performance of the baseline and Optuna-optimized models, which had the same test MSE of 2.188 and test R² of 0.857; hence, Optuna did not help to enhance the performance of the model. Skopt-optimized model slightly outperformed the baseline model in terms of training performance with Train MSE = 0.982, Train R² = 0.905, whereas the testing performance stayed the same. Such behaviour is due to the limited number of hyperparameters for the model. Implementation of 10-fold cross-validation helped to greatly improve the performance of the model. Training MSE was reduced to 0.301 and testing MSE was reduced to 0.610, which equals to 72.1% reduction in prediction error. The presence of similar values found for the baseline, Optuna, and skopt models through cross-validation proves that the effect of hyperparameter tuning is not significant in the case of Linear Regression. On the contrary, it is caused by using 10-fold cross-validation, which decreases the impact of train/test splitting bias, enabling each element to take part in the training and validation process. Furthermore, the similarity of performance on training and testing datasets suggests that the model is quite stable and not prone to overfitting.

**Table 9:** Linear Regression performance before and after cross-validation.

BEFORE CROSS VALIDATION											
MSE Values						R^2^ Values					
Without Optimizer		With Optuna Optimizer		With skOpt Optimizer		Without Optimizer		With Optuna Optimizer		With skOpt Optimizer	
Train	Test	Train	Test	Train	Test	Train	Test	Train	Test	Train	Test
2.18840	2.18840	2.18840	2.18840	0.98150	2.18840	0.85740	0.85740	0.85740	0.85740	0.90460	0.85740
**AFTER CROSS VALIDATION**											
**MSE Values**						**R**^**2**^ **Values**					
**Without Optimizer**		**With Optuna Optimizer**		**With skOpt Optimizer**		**Without Optimizer**		**With Optuna Optimizer**		**With skOpt Optimizer**	
**Train**	**Test**	**Train**	**Test**	**Train**	**Test**	**Train**	**Test**	**Train**	**Test**	**Train**	**Test**
0.30140	0.61030	0.30140	0.61030	0.30140	0.61030	0.97070	0.96020	0.97070	0.96020	0.97070	0.96020

#### Decision tree – before and after 10-fold cross-validation.

As observed from Table 10, the Decision Tree model has a testing MSE of 1.941 with an R² of 0.874 prior to the implementation of cross-validation. On the other hand, the Optimized model through Optuna resulted in almost the same result. Meanwhile, the skopt-optimized model had an almost perfect fit to the training data with a testing MSE of 0.008 and a train R² of 0.999 with no increase in testing performance, implying an increase in the complexity of the model. After applying the 10-fold cross-validation technique, the baseline model produced a testing MSE of 2.0645 and a testing R² of 0.8655, which is lower than the initial training and testing results. These results imply that the Decision Tree model is sensitive to the way the data is partitioned into training and testing sets and is prone to overfitting the training set. Meanwhile, the model optimized through Optuna produced even worse results since the testing MSE became 5.6368 while the testing R² became 0.6327, indicating underfitting. The skopt-optimized model had the same results as the baseline model after applying cross-validation. These results show that the Decision Tree model is more vulnerable to overfitting compared to the Linear Regression model. In addition, the hyperparameter tuning did not enhance the performance of the models, stressing the need for 10-fold cross-validation to evaluate the models’ performance.

**Table 10:** Decision Tree performance before and after cross validaton.

BEFORE CROSS VALIDATION											
MSE Values						R^2^ Values					
Without Optimizer		With Optuna Optimizer		With skOpt Optimizer		Without Optimizer		With Optuna Optimizer		With skOpt Optimizer	
Train	Test	Train	Test	Train	Test	Train	Test	Train	Test	Train	Test
1.94140	1.94140	1.94140	1.94140	0.00790	1.94140	0.87350	0.87350	0.87350	0.87350	0.99920	0.87350
**AFTER CROSS VALIDATION**											
**MSE Values**						**R**^**2**^ **Values**					
**Without Optimizer**		**With Optuna Optimizer**		**With skOpt Optimizer**		**Without Optimizer**		**With Optuna Optimizer**		**With skOpt Optimizer**	
**Train**	**Test**	**Train**	**Test**	**Train**	**Test**	**Train**	**Test**	**Train**	**Test**	**Train**	**Test**
0.00000	2.06450	1.26850	5.63680	0.00000	2.06450	1.00000	0.86550	0.87670	0.63270	1.00000	0.86550

#### Support vector regression – before and after 10-fold cross-validation.

As observed from results shown in Table 11, prior to cross-validation, the SVR model had poor prediction capabilities with the training and testing MSE of 13.3508 and R² value of 0.1300. This confirms that the default hyperparameters failed to create an accurate prediction model for the relationship between the process parameters and hardness. Optimization with Optuna gave only a marginal improvement in performance, with R² = 0.1947, while skopt optimization greatly improved it by reducing testing MSE to 1.3397 and R² to 0.9127. With 10-fold cross-validation, however, the predictive performance of all SVR models was improved. Testing MSE and R² of the default SVR model were reduced to 2.3876 and 0.8444, respectively, meaning that cross-validation improved the model performance estimation process. Optuna-optimized SVR further increased the performance through the testing MSE of 0.7066 and R² of 0.9540, which means that there is high prediction accuracy and low risk of overfitting. Skopt optimization gave the best results with the testing MSE of 0.2662 and R² of 0.9827. The overall results of the model show that the 10-fold cross-validation technique greatly contributed to the accuracy of the SVR analysis, while the optimization procedure greatly improved the model’s prediction. In terms of the SVR models, skopt SVR showed the best predictive accuracy, followed by the Optuna SVR, proving the efficiency of using hyperparameters for hardness prediction of FDM-produced PMMA samples.

**Table 11:** Support Vector Regression performance before and after cross-validation.

BEFORE CROSS VALIDATION											
MSE Values						R^2^ Values					
Without Optimizer		With Optuna Optimizer		With skOpt Optimizer		Without Optimizer		With Optuna Optimizer		With skOpt Optimizer	
Train	Test	Train	Test	Train	Test	Train	Test	Train	Test	Train	Test
13.35080	13.35080	12.35840	12.35840	0.66760	1.33970	0.13000	0.13000	0.19470	0.19470	0.93510	0.91270
**AFTER CROSS VALIDATION**											
**MSE Values**						**R**^**2**^ **Values**					
**Without Optimizer**		**With Optuna Optimizer**		**With skOpt Optimizer**		**Without Optimizer**		**With Optuna Optimizer**		**With skOpt Optimizer**	
**Train**	**Test**	**Train**	**Test**	**Train**	**Test**	**Train**	**Test**	**Train**	**Test**	**Train**	**Test**
0.71840	2.38760	0.33030	0.70660	0.00000	0.26620	0.93020	0.84440	0.96790	0.95400	1.00000	0.9827

#### AdaBoost - before and after 10-fold cross-validation.

It can be observed from Table 12 that without cross-validation, the AdaBoost model had an MSE of 3.251 for training and an MSE of 3.685 for testing, together with the training and testing R² value of 0.788 and 0.760, respectively. This shows that the baseline model had a moderate level of predictability. Optimizing through Optuna and skopt gave a slight improvement to the testing R² for the baseline, resulting in the values of 0.777 and 0.776, respectively. With the implementation of 10-fold cross-validation, all the AdaBoost models performed better regarding their predictive ability. For the baseline model, there was an improvement in both the MSE of 3.229 and the R² of 0.790, showing that cross-validation indeed reduced bias from the train-test split of the dataset. For the AdaBoost that was optimized using Optuna, the model was able to perform the best, with an MSE of 2.650 and a testing R² of 0.8273. Considering the skopt-optimized AdaBoost, it performed well with an MSE of 2.699 and a testing R² of 0.824. However, with a training R² of 0.977, it is evident that the skopt-optimized AdaBoost model can give a better fit to the training data compared to the testing data. Thus, the application of 10-fold cross-validation increased the robustness of AdaBoost model evaluation, while the use of Bayesian optimization increased its prediction accuracy. Out of all the optimized models, Optuna yielded a good compromise between the two metrics, while skopt yielded the best training accuracy but with slightly poorer testing results.

**Table 12:** AdaBoost performance before and after cross-validation.

BEFORE CROSS VALIDATION											
MSE Values						R^2^ Values					
Without Optimizer		With Optuna Optimizer		With skOpt Optimizer		Without Optimizer		With Optuna Optimizer		With skOpt Optimizer	
Train	Test	Train	Test	Train	Test	Train	Test	Train	Test	Train	Test
3.25110	3.68520	3.49750	3.42030	0.84480	3.44070	0.78810	0.75990	0.77210	0.77710	0.91790	0.77580
**AFTER CROSS VALIDATION**											
**MSE Values**						**R**^**2**^ **Values**					
**Without Optimizer**		**With Optuna Optimizer**		**With skOpt Optimizer**		**Without Optimizer**		**With Optuna Optimizer**		**With skOpt Optimizer**	
**Train**	**Test**	**Train**	**Test**	**Train**	**Test**	**Train**	**Test**	**Train**	**Test**	**Train**	**Test**
0.85670	3.22870	0.69440	2.64950	0.23510	2.69890	0.91680	0.78960	0.93250	0.82730	0.97720	0.82410

#### XGBoost - before and after 10-fold cross-validation.

From Table 13, considering XGBoost Model, the initial baseline model showed outstanding performance, achieving a training and testing MSE of 0.445 with an associated R² of 0.971. Optimizing the model via Optuna optimization improved its predictive ability further by decreasing the MSE to 0.356 and increasing the R² to 0.977. The optimized using skopt showed nearly a perfect fit for the training dataset with Train MSE = 0.000; Train R² = 1.000, and the testing R² was 0.968, showing good performance but without any improvement over the Optuna-optimized model. After the application of 10-fold cross-validation, the performance of the XGBoost models did not change significantly. The baseline model showed a testing MSE of 0.938 and a testing R² of 0.939, thus demonstrating its good generalization capabilities even when subjected to a stricter validation scheme. The most effective among the XGBoost models optimized via Optuna optimization showed a testing MSE of 0.114 and a testing R² of 0.993. Likewise, the model optimized using skopt showed a testing MSE of 0.198 and a testing R² of 0.987. Thus the 10-fold cross-validation demonstrated the stability of the XGBoost model, while the application of Bayesian optimization greatly improved the prediction capability of this model. XGBoost with the optimization by Optuna attained the best prediction accuracy and the least prediction error, while the XGBoost with the optimization by skopt performed very well in terms of generalization.

**Table 13:** Linear regression performance before and after cross-validation.

BEFORE CROSS VALIDATION												
MSE Values							R^2^ Values					
Without Optimizer		With Optuna Optimizer			With skOpt Optimizer		Without Optimizer		With Optuna Optimizer		With skOpt Optimizer	
Train	Test	Train	Test		Train	Test	Train	Test	Train	Test	Train	Test
0.44470	**0.44470**	0.35550	0.35550		0.00000	0.48910	0.97100	**0.97100**	0.97680	0.97680	1.00000	0.96810
**AFTER CROSS VALIDATION**												
**MSE Values**							**R^2^ Values**					
**Without Optimizer**		**With Optuna Optimizer**		**With skOpt Optimizer**			**Without Optimizer**		**With Optuna Optimizer**		**With skOpt Optimizer**	
**Train**	**Test**	**Train**	**Test**	**Train**		**Test**	**Train**	**Test**	**Train**	**Test**	**Train**	**Test**
0.00000	0.93750	0.01300	0.11380	0.02580		0.19790	1.00000	0.93890	0.99870	0.99260	0.99750	0.98710

#### CatBoost – before and after 10-fold cross-validation.

Considering CatBoost model, from the results displayed in Table 14, prior to cross-validation, the performance of the model showed that it had training and testing MSE equal to 1.845 with an R² of 0.880. Optimization using Optuna made a great improvement in the model, providing a testing MSE of 0.082 and R² of 0.995, thus proving the efficiency of hyperparameter optimization through the Bayesian method. Similarly, the skopt-optimized model provided nearly perfect training performance with Training MSE = 0.005, Training R² = 1.000, as well as a testing R² of 0.971. The introduction of 10-fold cross-validation improved the performance of all CatBoost algorithms. The testing performance of the baseline CatBoost is shown to be equal to an MSE of 1.764 and an R² of 0.885, thus showing that the model generalizes well. The Optuna-optimized model showed the best results, providing the lowest testing MSE of 0.138 and the highest testing R² of 0.991. Similar results were obtained from the skopt-optimized model, which has a testing MSE of 0.218 and an R² of 0.986. Thus, the outcomes reveal that the 10-fold cross-validation approach offers a more accurate evaluation of CatBoost performance while Bayesian optimization significantly boosts prediction capacity. The Optuna-optimized CatBoost had the best prediction accuracy and the least prediction error among all the variants of CatBoost, but the skopt-optimized model was very robust and generalizable. Consequently, it can be stated that the CatBoost method, especially when optimized via Optuna, is very efficient in predicting the hardness of FDM-made PMMA samples.

**Table 14:** Linear regression performance before and after cross-validation.

BEFORE CROSS VALIDATION											
MSE Values						R^2^ Values					
Without Optimizer		With Optuna Optimizer		With skOpt Optimizer		Without Optimizer		With Optuna Optimizer		With skOpt Optimizer	
Train	Test	Train	Test	Train	Test	Train	Test	Train	Test	Train	Test
1.84540	1.84540	0.08160	**0.08160**	0.00530	**0.43970**	0.87970	0.87970	0.99470	**0.99470**	0.99950	**0.97130**
**AFTER CROSS VALIDATION**											
**MSE Values**						**R**^**2**^ **Values**					
**Without Optimizer**		**With Optuna Optimizer**		**With skOpt Optimizer**		**Without Optimizer**		**With Optuna Optimizer**		**With skOpt Optimizer**	
**Train**	**Test**	**Train**	**Test**	**Train**	**Test**	**Train**	**Test**	**Train**	**Test**	**Train**	**Test**
0.00010	1.76390	0.00500	0.13760	0.00000	0.21800	1.00000	0.88510	0.99950	0.99100	1.00000	0.98580

#### Model generalization and overfitting.

A few optimized ensemble learning algorithms resulted in very high training accuracies, in terms of training R² scores almost reaching unity. This would be a possible indication of an overfitting phenomenon, especially in relation to flexible nonlinear algorithms like XGBoost and CatBoost. To test whether this is the case, the models have been evaluated via 10-fold cross-validation, which is a stricter method of assessing prediction accuracy than a simple training and testing split. The findings from the 10-fold cross-validation test showed that the optimized models kept their high prediction accuracies when validated through independent folds. In particular, the Optuna-optimized XGBoost model was found to yield a training R² score of 0.9987, a testing R² score of 0.9926, and a cross-validation R² score of 0.9886 on average. Likewise, the Optuna-optimized CatBoost model generated a training R² score of 0.9995, a testing R² score of 0.9910, and a cross-validation R² score of 0.9858 on average. The differences in the performance between the two testing and cross-validation cases were below 1%. Further proof of the consistency of the predictions of the optimized models can be observed through the low standard deviations in cross-validation. Therefore, even though some of the models have almost perfect fits for the training observations, the predictions produced by the models are consistent when the models are validated using new data. The presence of nearly perfect training results is explained by two features of the current study. First, the experimental data were collected using a balanced full-factorial design (3⁴ = 81 experiments). As a result, there exists a strong correlation between the process parameters and hardness. The second reason for the presence of nearly perfect results is that the gradient boosting algorithms like XGBoost and CatBoost are highly representative models, and therefore, after tuning, can accurately predict deterministic nonlinear relationships. However, the results of the current study are based mainly on cross-validation, which reduces the chance of misinterpretation.

## Conclusion

PMMA, with its biocompatibility, is being widely used for medical applications. The influence of process parameters in an additively fabricated PMMA for biomedical applications is established in this research article. Machine learning models were used to predict the hardness of the 3D-printed part. Linear regression, decision tree, support vector regression, XGBoost, CatBoost, and AdaBoost were the six machine learning models used in this research to predict hardness. XGBoost model was the best-fit model initially predicting the hardness of 3D printed parts with minimal errors. However, when optimization techniques were used, CatBoost outperformed other models to become the best-fit model. Optuna and skOpt were the two optimizers considered for this investigation. CatBoost has performed well with both optimizers, confirming the use of optimizers to improve the model’s performance. The results from the investigation confirm that the CatBoost model could be effectively used to predict the hardness of 3D-printed PMMA parts for biomedical applications with the help of Optuna or skOpt optimizers. When no optimizers are used, the XGBoost model must be used to effectively predict the hardness of additively fabricated PMMA parts. Thus, the investigation provides a feasible solution for medical experts to predict the hardness of biomedical parts 3D printed with PMMA materials. This would help them avoid the dependence on mechanical testing methods to determine the hardness of parts. Apart from this, it is found from the investigation that the hardness of additively printed PMMA parts increases with a decrease in layer height. A layer height of 0.1 mm has produced maximum hardness. For a 3D-printed part to be hard enough to withstand external forces, it should be filled with material, which indicates the impact of infill density. A 3D-printed part with 100% infill density results in higher hardness, enhancing the part’s ability to withstand external forces. Similarly, the Lines infill pattern produces higher hardness than the Cubic or trihexagonal infill pattern. Raster orientations of 0°, 45° and 90° were considered in this investigation, of which 0° raster orientation produced the maximum hardness. Thus, this research investigation would help medical experts achieve the required hardness with 3D-printed PMMA parts for biomedical applications.

## Supporting information

S1 File Appendices: Dataset used in the research. (DOC)
