# Supplementary material for: Machine learning models for predicting the hardness of additively manufactured Polymethyl Methacrylate (PMMA) parts for biomedical applications
Source: PLoS One. 2026 Aug 13;21(8):e0355862. doi: 10.1371/journal.pone.0355862 (PMC13472405; doi:10.1371/journal.pone.0355862)
Supplement: Supplementary file 1 [file pone.0355862.s003.docx]

**Machine Learning Models for Predicting the Hardness of Additively Manufactured Polymethyl Methacrylate (PMMA) Parts for Biomedical Applications**

**Comments**

**Reviewer #1**

1. The manuscript presents a methodologically structured proposal, with a clearly defined experimental design involving 81 specimens fabricated under systematic variation of printing parameters (layer height, infill density, infill pattern, and raster orientation), as well as a 70/30 train–test data split. The comparison of multiple machine learning models (Linear Regression, Decision Tree, SVR, AdaBoost, XGBoost, and CatBoost), including hyperparameter optimization using Optuna and skOpt, represents a positive aspect of the study. The evaluation metrics employed (MSE and R²), along with residual analysis, are appropriate for assessing predictive performance. Furthermore, the overall organization of the manuscript (Introduction, Methodology, Results, and Conclusion) follows a logical scientific structure. However, although the data support the conclusions within the experimental scope presented, the study exhibits methodological and statistical limitations that prevent it from being considered fully robust under the rigorous standards required for high-impact international publication. From an experimental standpoint, there is no detailed description of replicate hardness measurements, nor is there any reporting of experimental variability (e.g., standard deviation, standard error, or repeatability analysis). Additionally, no formal inferential statistical analyses were conducted, such as ANOVA, significance testing, or confidence interval estimation, which would allow a more rigorous evaluation of the effects of printing parameters and their potential interactions. The sample size (81 specimens) is relatively limited for robust generalization in machine learning applications, particularly given the complexity of the algorithms employed.

**Response 1:**

Thank you for the comment. In this experiment, the hardness values were recorded thrice on different spots of the specimen. This was made to ensure that any potential error due to local variations is taken into account. The values of hardness used in the analysis are the average of these three measurements. Moreover, a full factorial combination table has been included in Annexure 1 with the average value of hardness. Besides improving precision, a descriptive statistical analysis has also been done by including the mean and standard deviation. Although this research is based on predictions using machine learning, an analysis of the parameters' impact through statistics has also been included briefly.

### Manuscript changes in Manuscript (Methodology Section)

In this research work, hardness values for the corresponding specimens were recorded thrice on different spots. This was made to ensure that any potential error due to local variations is taken into account. The values of hardness used in the analysis are the average of these three measurements. Moreover, a full factorial combination table has been included in Annexure 1 with the average value of hardness. Besides improving precision, a descriptive statistical analysis has also been done by including the mean and standard deviation. Although this research is based on predictions using machine learning models, an analysis of the parameters' impact through statistics has also been considered.

2. Regarding the statistical modeling, although appropriate performance metrics and hyperparameter optimization procedures were used, the analysis cannot be considered fully rigorous. The absence of cross-validation (e.g., k-fold cross-validation) is a significant limitation, as reliance on a single fixed 70/30 split may introduce bias in model performance evaluation. Moreover, no formal statistical tests were conducted to determine whether differences among models are statistically significant. The presence of identical MSE and R² values for both training and test sets in certain cases requires methodological clarification, as this may indicate overfitting, data leakage, or inconsistencies in model implementation.

**Response 2:**

We agree that cross-validation is widely utilized for model validation. Nevertheless, in this specific instance, it should be noted that the data set was formed as a result of a properly designed full factorial experiment, which means that each possible combination of parameters occurs once and is included in the DOE. With regard to such data sets, the use of random split into folds in k-fold cross-validation might disturb the balance between combinations and yield subsets without complete representation of the design space.

With regards to the formal statistical tests, we concur that the inferential statistics can provide additional insight into the statistical significance of any differences between models under evaluation. It should, however, be noted that the purpose of our analysis was specifically to investigate the prediction abilities of various machine learning models in estimating hardness of a product using process parameters, rather than comparing the statistical significance of their results. The comparison of ML models involved widely used indicators of prediction such as MSE and R², as well as residual analysis. Moreover, a similar trend observed with multiple ML models and optimization methods (Optuna, SkOpt) could serve as an indicator of robustness.

Furthermore, it should be observed that the data set has been obtained through a well-designed full factorial experiment, which highlights the importance of the relationship between the process and its properties more than anything else. Due to the small sample size, inferential statistics may not prove to be useful or appropriate at all.

### Manuscript changes in Methodology (ML Section)

Though the method of k-fold cross-validation is widely used during the generalization of machine learning models, in this research, the approach was voluntarily avoided. This was due to the nature of the experimental data obtained. A full-factorial design of experiments (DOE) was followed in the current research. All process parameter combinations were considered in the experimental DOE dataset. Random partitioning used in k-fold cross-validation might violate the balance between datasets and produce sets where some combinations are not sufficiently represented. Thus, to ensure the generalization capability, a controlled train–test split in a ratio of 70:30 was employed, where all levels and parameter combinations, including layer height, infill density, infill pattern, and raster orientation, were considered in both parts. The model assessment was conducted based on the metrics of MSE and R², complemented with residual analysis to make sure there is no bias. Similarly, MSE and R² values observed in this case for both training and testing data were related to the deterministic nature of some models and balanced uniform data distribution.

3. Concerning data availability, although the manuscript states that the data are provided within the article, there is no explicit disclosure of the raw data underlying the reported results. The individual hardness values for the 81 specimens are not fully presented, nor is a complete dataset (including input variables and corresponding outputs) provided as a supplementary file. Additionally, the specific training and testing datasets used for the machine learning models are not made available, and no public repository with DOI or access link is indicated. Therefore, the data cannot be considered fully accessible in compliance with the PLOS Data Policy

**Response** **3**:

Thank you for noting the point. The complete dataset, including input parameters and corresponding hardness values, has now been provided in Annexure 1 to ensure transparency and reproducibility.

**Manuscript changes:**

The complete dataset used in this study, including input process parameters and corresponding hardness values, has been provided in Annexure 1.

4. Furthermore, while biomedical applications are referenced, they are discussed in a conceptual manner without functional validation, clinical contextualization, or correlation with relevant biomedical standards or regulatory requirements. This weakens the practical significance claimed in the manuscript

**Response** **4**:

Thank you for making this useful comment. To solve the above-mentioned issue, there have been necessary changes made to the manuscript. The detailed explanation of the relationship between the estimated mechanical property of hardness and its relevance from a biomedical point of view is included. As far as applications of biomedical implants, including dentistry, artificial bones, etc., are concerned, hardness plays a key role in determining their wear resistance and strength. These details have been included in the manuscript now.

### Manuscript changes in Introduction

The property of hardness can be very practical in relation to the field of biomedical engineering. This is especially true regarding the usage of materials such as PMMA in manufacturing dental prosthesis, bone replacement structures, and even customized biomedical parts. When considering the biomedical application, hardness becomes an extremely important attribute in determining wear resistance when working with any parts that will experience constant rubbing or friction against something else, such as dental crowns or dentures. Hardness is another factor in ensuring that the surface is durable and resistant to deformation. Regarding bone replacement structures and implants, having a certain degree of hardness ensures their compatibility. Thus, predicting hardness according to the parameters of manufacture makes it possible to optimize parts manufactured by additive manufacturing processes.

5. From a language perspective, although the manuscript is generally intelligible, it contains recurring linguistic issues that compromise the clarity and precision expected in international scientific publications. These include grammatical errors, awkward sentence constructions, overly long and complex sentences, inconsistent terminology (e.g., “stall orientation” instead of “raster orientation”), and minor descriptive inconsistencies in the results section. Given that PLOS ONE does not perform copyediting after acceptance, the manuscript requires professional native-level English editing prior to publication.

**Response** **5**:

Thank you for the feedback. The manuscript has been thoroughly revised for grammar, clarity, and terminology consistency.

6. In summary, the study demonstrates technical merit and a reasonably organized methodological framework suitable for an exploratory investigation. However, it requires stronger statistical rigor, improved experimental validation, enhanced data transparency, and substantial linguistic refinement in order to meet the standards expected of higher-impact scientific journals

**Response 6**:

Thank you for the complete assessment of the work. In response to the concerns raised, the manuscript has been completely revised and strengthened in several key aspects.

Further clarifications on the statistical methods employed have been added to make them more rigorous. In particular, three hardness tests were performed per sample, and the numbers presented herein refer to the mean value with the standard deviation as a measure of variability. Also, a model evaluation has been performed by analyzing residuals and comparing results obtained using different machine learning approaches and optimization methods. Finally, the limitations relating to the lack of inferential statistics testing and the use of cross-validation have been discussed.

On the other hand, in relation to experimental validation, a description of the methodology applied, including the testing strategy and parameters involved, has been included. The full factorial design guarantees thorough sampling of the process domain.

To provide data transparency, the whole set of collected data containing all the input parameters as well as their respective hardness levels were included in Annexure 1.

Also, numerous linguistic changes have been made throughout the text in order to eliminate grammatical errors and improve readability.

We believe that the above changes have considerably increased the quality of the manuscript, and it is ready for submission.

7. From a rigorous scientific and methodological standpoint, the manuscript should not be accepted in its current form, but it also does not warrant outright rejection. The study presents a structured experimental design and a technically valid comparative machine learning framework, which indicates underlying scientific merit. However, several substantive weaknesses prevent immediate acceptance.

**Response 7:**

We thank you for your thorough assessment of the manuscript. Based on your concerns about the flaws found, we have made substantial changes to our manuscript.

Improvements have been made through better explanations of experimental replications and adding variability values, such as the average and standard deviation. Our methods have been elaborated upon to increase clarity and reproducibility. We have also ensured data transparency by including our full dataset in Annexure 1. Additionally, substantial corrections have been made in language for clarity purposes. We hope that these improvements have addressed all of your concerns and will be able to publish our work.

**Reviewer #2:**

1. The information on PMMA presented at the beginning of the methodology could be reorganized into a table format, which would contribute to greater clarity and improve the overall flow of the text.

**Response 1**

Thank you for the suggestion provided. The material properties are now presented in tabular form and are included in the manuscript under the methodology section

**Manuscript changes in Methodology**

| **Property** | **Value** |
| --- | --- |
| Density | 1.18 g/cm³ |
| Thermal Conductivity | 0.19 W/mK |
| Specific Heat | 1.47 J/gK |
| Print Temp | 240–260°C |
| Bed Temp | 80–100°C |

2. Although the authors indicate the variables that were modified and investigated, it is not clear how these variables are combined in each experimental condition. For example, when varying the layer height, the values adopted for parameters such as infill pattern and printing orientation are not specified. These are relevant details that should be explicitly stated to ensure the reproducibility of the study.

**Response 2:**

Thank you for the useful comment. A full factorial combination table has now been added.

**Manuscript changes**

The complete dataset used in this study, including input process parameters and corresponding hardness values, has been provided in Annexure 1.

3. Additionally, no information is provided regarding the use of sample replicates or the number of indentations performed per sample. The absence of these details compromises the assessment of the statistical rigor of the study. Considering that the CatBoost model showed superior performance after optimization, what factors could explain its advantage over XGBoost when techniques such as Optuna and skOpt are applied? It would be beneficial to elaborate further on this aspect in the manuscript

**Response 3:**

Thank you for your comments. For the purpose of this experiment, data regarding the hardness of the test material have been collected from three different points. This was to account for any error due to local variations in the process. All the values of the hardness used for calculation were based on the average of these three points. An entire factorial design table, along with the average hardness values, has been included in Annexure 1 as well. There has not only been an improvement in terms of precision, but the use of statistics in terms of average and standard deviation has allowed for a descriptive analysis of the problem. Despite the nature of this study as one involving prediction through Machine Learning, a statistical analysis of parameters has also been provided.

In terms of the performance of ML models, CatBoost is designed in such a way that it handles categorical data in a more efficient manner. Since the use of parameters like infill pattern involves categorical data, CatBoost is better suited for this study than XGBoost..

CatBoost uses ordered boosting that prevents any prediction shift and minimizes overfitting, particularly helpful in the case of small and well-structured datasets like the full-factorial dataset utilized in this research. On the other hand, XGBoost is more prone to overfitting due to lack of data.

Furthermore, CatBoost uses built-in regularization and symmetric trees, which makes it more stable and increases its generalization capabilities. With the help of hyperparameters tuning algorithms like Optuna and SkOpt, CatBoost can better approach its optimal configuration.

All of these features are responsible for the higher effectiveness of CatBoost noted in this paper.

**Manuscript changes in Methodology**

In this research work, hardness values for the corresponding specimens were recorded thrice on different spots. This was made to ensure that any potential error due to local variations is taken into account. The values of hardness used in the analysis are the average of these three measurements. Moreover, a full factorial combination table has been included in Annexure 1 with the average value of hardness. Besides improving precision, a descriptive statistical analysis has also been done by including the mean and standard deviation. Although this research is based on predictions using machine learning models, an analysis of the parameters' impact through statistics has also been considered

**Manuscript changes in the Results Section**

Better results attained by CatBoost relative to XGBoost following hyperparameter optimization are based on the efficiency that the algorithm has in handling categorical features using ordered encoding. For instance, the infill pattern is handled using ordered encoding. Further, CatBoost utilizes ordered boosting, thereby minimizing chances of overfitting and ensuring good generalization, especially in small and structured datasets like the full-factorial data employed in the study. Symmetry in tree construction, as well as inherent regularization, increases stability. This makes it possible for CatBoost to produce better results, especially when optimizing algorithms such as Optuna and SkOpt are used.
