# Supplementary material for: Machine learning models for predicting the hardness of additively manufactured Polymethyl Methacrylate (PMMA) parts for biomedical applications
Source: PLoS One. 2026 Aug 13;21(8):e0355862. doi: 10.1371/journal.pone.0355862 (PMC13472405; doi:10.1371/journal.pone.0355862)
Supplement: Supplementary file 3 [file pone.0355862.s001.doc]

**Appendices: Dataset used in the research**

| **Specimen No** | **Input Process Parameters** | | | | **Measured Values** | | | **Average HV** |
| --- | --- | --- | --- | --- | --- | --- | --- | --- |
|  | **Layer Height** | **Infill density** | **Infill Pattern** | **Raster Orientation** | **HV 1** | **HV 2** | **HV 3** |  |
| 1 | 0.1mm | 50% | Lines | 0° | 87.8 | 82.77 | 87.01 | 85.86 |
| 2 |  |  |  | 45° | 79.19 | 84.65 | 88.88 | 84.24 |
| 3 |  |  |  | 90° | 79.65 | 85.11 | 89.34 | 84.7 |
| 4 |  |  | Cubic | 0° | 77.23 | 82.69 | 86.92 | 82.28 |
| 5 |  |  |  | 45° | 73.75 | 79.21 | 83.44 | 78.8 |
| 6 |  |  |  | 90° | 75.3 | 80.76 | 84.99 | 80.35 |
| 7 |  |  | TriHexagonal | 0° | 79.22 | 84.68 | 88.91 | 84.27 |
| 8 |  |  |  | 45° | 76.24 | 81.7 | 85.93 | 81.29 |
| 9 |  |  |  | 90° | 76.65 | 82.11 | 86.34 | 81.7 |
| 10 |  | 75% | Lines | 0° | 83.35 | 88.81 | 93.05 | 88.4 |
| 11 |  |  |  | 45° | 78.81 | 88.77 | 93.01 | 86.86 |
| 12 |  |  |  | 90° | 79.27 | 89.23 | 93.47 | 87.32 |
| 13 |  |  | Cubic | 0° | 76.85 | 86.81 | 91.05 | 84.9 |
| 14 |  |  |  | 45° | 73.37 | 83.33 | 87.57 | 81.42 |
| 15 |  |  |  | 90° | 74.92 | 84.88 | 89.12 | 82.97 |
| 16 |  |  | TriHexagonal | 0° | 78.84 | 88.8 | 93.04 | 86.89 |
| 17 |  |  |  | 45° | 78.36 | 83.82 | 88.06 | 83.41 |
| 18 |  |  |  | 90° | 78.77 | 84.23 | 88.47 | 83.82 |
| 19 |  | 100% | Lines | 0° | 86.23 | 91.69 | 95.93 | 91.28 |
| 20 |  |  |  | 45° | 84.23 | 89.69 | 93.93 | 89.28 |
| 21 |  |  |  | 90° | 84.73 | 90.19 | 94.43 | 89.78 |
| 22 |  |  | Cubic | 0° | 82.23 | 87.69 | 91.93 | 87.28 |
| 23 |  |  |  | 45° | 76.23 | 86.19 | 90.43 | 84.28 |
| 24 |  |  |  | 90° | 78.23 | 88.19 | 92.43 | 86.28 |
| 25 |  |  | TriHexagonal | 0° | 81.73 | 91.69 | 95.93 | 89.78 |
| 26 |  |  |  | 45° | 78.73 | 88.69 | 92.93 | 86.78 |
| 27 |  |  |  | 90° | 79.23 | 89.19 | 93.43 | 87.28 |
| 28 | 0.2mm | 50% | Lines | 0° | 77.81 | 87.77 | 92 | 85.86 |
| 29 |  |  |  | 45° | 78.27 | 83.73 | 87.96 | 83.32 |
| 30 |  |  |  | 90° | 78.73 | 84.19 | 88.42 | 83.78 |
| 31 |  |  | Cubic | 0° | 76.31 | 81.77 | 86 | 81.36 |
| 32 |  |  |  | 45° | 72.83 | 78.29 | 82.52 | 77.88 |
| 33 |  |  |  | 90° | 74.38 | 79.84 | 84.07 | 79.43 |
| 34 |  |  | TriHexagonal | 0° | 78.3 | 83.76 | 87.99 | 83.35 |
| 35 |  |  |  | 45° | 72.82 | 82.78 | 87.01 | 80.87 |
| 36 |  |  |  | 90° | 73.23 | 83.19 | 87.42 | 81.28 |
| 37 |  | 75% | Lines | 0° | 80.85 | 90.81 | 95.05 | 88.9 |
| 38 |  |  |  | 45° | 78.81 | 88.77 | 93.01 | 86.86 |
| 39 |  |  |  | 90° | 79.27 | 89.23 | 93.47 | 87.32 |
| 40 |  |  | Cubic | 0° | 76.85 | 86.81 | 91.05 | 84.9 |
| 41 |  |  |  | 45° | 75.87 | 81.33 | 85.57 | 80.92 |
| 42 |  |  |  | 90° | 77.42 | 82.88 | 87.12 | 82.47 |
| 43 |  |  | TriHexagonal | 0° | 81.34 | 86.8 | 91.04 | 86.39 |
| 44 |  |  |  | 45° | 78.36 | 83.82 | 88.06 | 83.41 |
| 45 |  |  |  | 90° | 78.77 | 84.23 | 88.47 | 83.82 |
| 46 |  | 100% | Lines | 0° | 84.98 | 90.44 | 94.68 | 90.03 |
| 47 |  |  |  | 45° | 82.94 | 88.4 | 92.64 | 87.99 |
| 48 |  |  |  | 90° | 83.4 | 88.86 | 93.1 | 88.45 |
| 49 |  |  | Cubic | 0° | 80.98 | 86.44 | 90.68 | 86.03 |
| 50 |  |  |  | 45° | 77.5 | 82.96 | 87.2 | 82.55 |
| 51 |  |  |  | 90° | 79.05 | 84.51 | 88.75 | 84.1 |
| 52 |  |  | TriHexagonal | 0° | 82.97 | 88.43 | 92.67 | 88.02 |
| 53 |  |  |  | 45° | 79.99 | 85.45 | 89.69 | 85.04 |
| 54 |  |  |  | 90° | 80.4 | 85.86 | 90.1 | 85.45 |
| 55 | 0.3mm | 50% | Lines | 0° | 78.19 | 83.64 | 87.88 | 83.24 |
| 56 |  |  |  | 45° | 76.15 | 81.6 | 85.84 | 81.2 |
| 57 |  |  |  | 90° | 76.61 | 82.06 | 86.3 | 81.66 |
| 58 |  |  | Cubic | 0° | 74.19 | 79.64 | 83.88 | 79.24 |
| 59 |  |  |  | 45° | 70.71 | 76.16 | 80.4 | 75.76 |
| 60 |  |  |  | 90° | 72.26 | 77.71 | 81.95 | 77.31 |
| 61 |  |  | TriHexagonal | 0° | 76.18 | 81.63 | 85.87 | 81.23 |
| 62 |  |  |  | 45° | 73.2 | 78.65 | 82.89 | 78.25 |
| 63 |  |  |  | 90° | 73.61 | 79.06 | 83.3 | 78.66 |
| 64 |  | 75% | Lines | 0° | 80.31 | 85.77 | 90 | 85.36 |
| 65 |  |  |  | 45° | 78.27 | 83.73 | 87.96 | 83.32 |
| 66 |  |  |  | 90° | 78.73 | 84.19 | 88.42 | 83.78 |
| 67 |  |  | Cubic | 0° | 76.31 | 81.77 | 86 | 81.36 |
| 68 |  |  |  | 45° | 72.83 | 78.29 | 82.52 | 77.88 |
| 69 |  |  |  | 90° | 74.38 | 79.84 | 84.07 | 79.43 |
| 70 |  |  | TriHexagonal | 0° | 78.3 | 83.76 | 87.99 | 83.35 |
| 71 |  |  |  | 45° | 75.32 | 80.78 | 85.01 | 80.37 |
| 72 |  |  |  | 90° | 75.73 | 81.19 | 85.42 | 80.78 |
| 73 |  | 100% | Lines | 0° | 84.06 | 89.52 | 93.76 | 89.11 |
| 74 |  |  |  | 45° | 79.52 | 89.48 | 93.72 | 87.57 |
| 75 |  |  |  | 90° | 79.98 | 89.94 | 94.18 | 88.03 |
| 76 |  |  | Cubic | 0° | 77.56 | 87.52 | 91.76 | 85.61 |
| 77 |  |  |  | 45° | 74.08 | 84.04 | 88.28 | 82.13 |
| 78 |  |  |  | 90° | 75.63 | 85.59 | 89.83 | 83.68 |
| 79 |  |  | TriHexagonal | 0° | 79.55 | 89.51 | 93.75 | 87.6 |
| 80 |  |  |  | 45° | 79.07 | 84.53 | 88.77 | 84.12 |
| 81 |  |  |  | 90° | 79.48 | 84.94 | 89.18 | 84.53 |
