# Supplementary material for: Machine learning models for predicting the hardness of additively manufactured Polymethyl Methacrylate (PMMA) parts for biomedical applications
Source: PLoS One. 2026 Aug 13;21(8):e0355862. doi: 10.1371/journal.pone.0355862 (PMC13472405; doi:10.1371/journal.pone.0355862)
Supplement: Supplementary file 2 [file pone.0355862.s004.docx]

**RESPONSE TO REVIEWER COMMENTS**

**REVIEWER #1:**

**COMMENT 1:**

The authors did not perform: ANOVA; statistical significance testing; confidence interval analysis; formal interaction effect analysis. Instead, they mainly justified the absence of these methods. From a scientific perspective, this considerably weakens the robustness of the experimental interpretation. For a full-factorial DOE, the absence of ANOVA is particularly problematic, since this type of experimental design is specifically intended for evaluating parameter effects and interactions.

**RESPONSE 1:**

We thank the reviewer for providing the valuable suggestion. A complete statistical analysis has been carried out in the revised manuscript.

The following analyses have been reported:

- ANOVA test with full factorial design for testing the statistical significance of all four printing parameters.
- ANOVA test with Interaction design for testing the statistical significance of interaction between the process parameters.
- 95% Confidence Interval Analysis for the hardness data.
- Interaction Effect Analysis along with Interaction plots.
- Statistical significance was tested at 95% confidence level.

The results obtained from ANOVA tests show that there is a significant effect of layer height, infill density, shell thickness, and print orientation on the hardness. Several interaction terms are also statistically significant.

The ANOVA tables, interaction effect analysis, confidence interval analysis, and discussions are included in the revised manuscript as a separate section entitled “**ANOVA Analysis**”

Thereby, the statistical analysis of experimental results has been significantly improved.

**COMMENT 2:**

The justification provided for not using k-fold cross-validation is not entirely convincing. For small datasets, cross-validation is generally recommended precisely to reduce train-test split bias. Moreover, several alternative approaches could have been considered, including: stratified validation; repeated k-fold validation; leave-one-out validation; grouped cross-validation. Although the explanation may be acceptable during revision, it remains methodologically weak.

**RESPONSE 2:**

We thank the reviewer for this important observation.

On the basis of this comment, the machine learning algorithm has been revised using 10-fold cross-validation for each machine learning model examined.

The new manuscript now contains the following information:

- 10-Fold Cross Validation approach
- Cross Validation MSE
- Cross Validation R²
- Comparison of the initial train-test method and 10-fold cross validation
- Generalization capability discussion

The cross-validation has shown that the optimized models keep their high predictive capabilities even when applied to new data. The close coincidence of the performance in the test method and in the cross-validation proves that the developed models have good generalization capability.

A new section "**Comparative Performance of Models after cross validation**" has been introduced into the manuscript.

**COMMENT 3:**

Several statistically questionable results are still present. For example: multiple models exhibit exactly identical training and testing metrics; some cases report R² = 1.0000; XGBoost optimization reports MSE = 0.0000. Such results are highly unusual for real experimental datasets. Even though the authors attempted to justify these findings based on balanced data distribution, there are still strong indications of: overfitting; data leakage; or excessive dataset simplification. This issue would still require deeper methodological clarification.

**RESPONSE 3:**

We appreciate the reviewer's careful examination of the reported machine learning results and bringing it into our notice.

In response to this comment, we critically analyzed all aspects of the machine learning pipeline, from data preparation to model building, optimization, and validation. A number of key points have been included into the revised version of the manuscript. The presented R² ≈ 1.0000 and MSE ≈ 0.0000 values refer to training-set results only for highly adaptive ensemble methods following Bayesian hyperparameter optimization procedure. This data was not used to evaluate the predictive ability of the models. In order to check whether the achieved results could be the consequence of overfitting or data leakage, we used 10-fold cross-validation procedure for all of the developed models. The outcomes of the cross-validation prove that the optimized models provide high performance on unseen data.

The differences between testing performance and cross-validation performance are small, indicating excellent model robustness and limited evidence of severe overfitting. Additionally, the complete preprocessing pipeline was re-examined to eliminate potential data leakage. Hyperparameter optimization and model fitting were performed exclusively using the training data within each validation fold, while the validation fold remained completely unseen during training. Thus the discussion has been revised to emphasize testing performance and cross-validation performance rather than training metrics, since these provide a more realistic assessment of model generalization. Accordingly, we believe that the additional validation significantly strengthens the statistical credibility of the proposed machine learning framework. This explanation has been added in the manuscript as a separate section entitled “**Model Generalization and Overfitting**”
