# Supplementary material for: Machine learning models for predicting the hardness of additively manufactured Polymethyl Methacrylate (PMMA) parts for biomedical applications
Source: PLoS One. 2026 Aug 13;21(8):e0355862. doi: 10.1371/journal.pone.0355862 (PMC13472405; doi:10.1371/journal.pone.0355862)
Supplement: Peer review history

# Peer review: Decision Letter 0

**DOI:** 10.1371/journal.pone.0355862.r001

**Authors:** André Gustavo Galdino

30 Mar 2026

Dear Dr. Katiyar,

Thank you for submitting your manuscript to PLOS ONE. After careful consideration, we feel that it has merit but does not fully meet PLOS ONE’s publication criteria as it currently stands. Therefore, we invite you to submit a revised version of the manuscript that addresses the points raised during the review process.

Please read reviewers' comments and improve your manuscript according to their suggestions.

Please submit your revised manuscript by May 14 2026 11:59PM. If you will need more time than this to complete your revisions, please reply to this message or contact the journal office at plosone@plos.org. When you're ready to submit your revision, log on to https://www.editorialmanager.com/pone/ and select the 'Submissions Needing Revision' folder to locate your manuscript file.

- A letter that responds to each point raised by the academic editor and reviewer(s). You should upload this letter as a separate file labeled 'Response to Reviewers'.
- A marked-up copy of your manuscript that highlights changes made to the original version. You should upload this as a separate file labeled 'Revised Manuscript with Track Changes'.
- An unmarked version of your revised paper without tracked changes. You should upload this as a separate file labeled 'Manuscript'.

If you would like to make changes to your financial disclosure, please include your updated statement in your cover letter. Guidelines for resubmitting your figure files are available below the reviewer comments at the end of this letter.

If applicable, we recommend that you deposit your laboratory protocols in protocols.io to enhance the reproducibility of your results. Protocols.io assigns your protocol its own identifier (DOI) so that it can be cited independently in the future. For instructions see: https://journals.plos.org/plosone/s/submission-guidelines#loc-laboratory-protocols. Additionally, PLOS ONE offers an option for publishing peer-reviewed Lab Protocol articles, which describe protocols hosted on protocols.io. Read more information on sharing protocols at https://plos.org/protocols?utm_medium=editorial-email&utm_source=authorletters&utm_campaign=protocols.

As the corresponding author, your ORCID iD is verified in the submission system and will appear in the published article. PLOS supports the use of ORCID, and we encourage all coauthors to register for an ORCID iD and use it as well. Please encourage your coauthors to verify their ORCID iD within the submission system before final acceptance, as unverified ORCID iDs will not appear in the published article. *Only*  the individual author can complete the verification step; PLOS staff *cannot*  verify ORCID iDs on behalf of authors.

We look forward to receiving your revised manuscript.

Kind regards,

André Gustavo de Sousa Galdino

Academic Editor

PLOS One

Journal Requirements:

When submitting your revision, we need you to address these additional requirements.

1. Please ensure that your manuscript meets PLOS ONE's style requirements, including those for file naming. The PLOS ONE style templates can be found at

https://journals.plos.org/plosone/s/file?id=wjVg/PLOSOne_formatting_sample_main_body.pdf and https://journals.plos.org/plosone/s/file?id=ba62/PLOSOne_formatting_sample_title_authors_affiliations.pdf

2. Please note that PLOS One has specific guidelines on code sharing for submissions in which author-generated code underpins the findings in the manuscript. In these cases, we expect all author-generated code to be made available without restrictions upon publication of the work. Please review our guidelines at https://journals.plos.org/plosone/s/materials-and-software-sharing#loc-sharing-code and ensure that your code is shared in a way that follows best practice and facilitates reproducibility and reuse.

3. Please update your submission to use the PLOS LaTeX template. The template and more information on our requirements for LaTeX submissions can be found at http://journals.plos.org/plosone/s/latex.

4. We note that Figure 1 in your submission contain copyrighted images. All PLOS content is published under the Creative Commons Attribution License (CC BY 4.0), which means that the manuscript, images, and Supporting Information files will be freely available online, and any third party is permitted to access, download, copy, distribute, and use these materials in any way, even commercially, with proper attribution. For more information, see our copyright guidelines: http://journals.plos.org/plosone/s/licenses-and-copyright.

We require you to either (1) present written permission from the copyright holder to publish these figures specifically under the CC BY 4.0 license, or (2) remove the figures from your submission:

a. You may seek permission from the original copyright holder of Figure 1 to publish the content specifically under the CC BY 4.0 license.

We recommend that you contact the original copyright holder with the Content Permission Form (http://journals.plos.org/plosone/s/file?id=7c09/content-permission-form.pdf) and the following text:

“I request permission for the open-access journal PLOS ONE to publish XXX under the Creative Commons Attribution License (CCAL) CC BY 4.0 (http://creativecommons.org/licenses/by/4.0/). Please be aware that this license allows unrestricted use and distribution, even commercially, by third parties. Please reply and provide explicit written permission to publish XXX under a CC BY license and complete the attached form.”

Please upload the completed Content Permission Form or other proof of granted permissions as an "Other" file with your submission.

In the figure caption of the copyrighted figure, please include the following text: “Reprinted from [ref] under a CC BY license, with permission from [name of publisher], original copyright [original copyright year].”

b. If you are unable to obtain permission from the original copyright holder to publish these figures under the CC BY 4.0 license or if the copyright holder’s requirements are incompatible with the CC BY 4.0 license, please either i) remove the figure or ii) supply a replacement figure that complies with the CC BY 4.0 license. Please check copyright information on all replacement figures and update the figure caption with source information. If applicable, please specify in the figure caption text when a figure is similar but not identical to the original image and is therefore for illustrative purposes only.

5. If the reviewer comments include a recommendation to cite specific previously published works, please review and evaluate these publications to determine whether they are relevant and should be cited. There is no requirement to cite these works unless the editor has indicated otherwise.

[Note: HTML markup is below. Please do not edit.]

Reviewers' comments:

Reviewer's Responses to Questions

**Comments to the Author**

1. Is the manuscript technically sound, and do the data support the conclusions?

Reviewer #1: Partly

Reviewer #2: Yes

**********

2. Has the statistical analysis been performed appropriately and rigorously?

Reviewer #1: No

Reviewer #2: No

**********

3. Have the authors made all data underlying the findings in their manuscript fully available?

The PLOS Data policy

Reviewer #1: No

Reviewer #2: No

**********

4. Is the manuscript presented in an intelligible fashion and written in standard English?

Reviewer #1: No

Reviewer #2: Yes

**********

Reviewer #1: The manuscript presents a methodologically structured proposal, with a clearly defined experimental design involving 81 specimens fabricated under systematic variation of printing parameters (layer height, infill density, infill pattern, and raster orientation), as well as a 70/30 train–test data split. The comparison of multiple machine learning models (Linear Regression, Decision Tree, SVR, AdaBoost, XGBoost, and CatBoost), including hyperparameter optimization using Optuna and skOpt, represents a positive aspect of the study. The evaluation metrics employed (MSE and R²), along with residual analysis, are appropriate for assessing predictive performance. Furthermore, the overall organization of the manuscript (Introduction, Methodology, Results, and Conclusion) follows a logical scientific structure.

However, although the data support the conclusions within the experimental scope presented, the study exhibits methodological and statistical limitations that prevent it from being considered fully robust under the rigorous standards required for high-impact international publication.

From an experimental standpoint, there is no detailed description of replicate hardness measurements, nor is there any reporting of experimental variability (e.g., standard deviation, standard error, or repeatability analysis). Additionally, no formal inferential statistical analyses were conducted, such as ANOVA, significance testing, or confidence interval estimation, which would allow a more rigorous evaluation of the effects of printing parameters and their potential interactions. The sample size (81 specimens) is relatively limited for robust generalization in machine learning applications, particularly given the complexity of the algorithms employed.

Regarding the statistical modeling, although appropriate performance metrics and hyperparameter optimization procedures were used, the analysis cannot be considered fully rigorous. The absence of cross-validation (e.g., k-fold cross-validation) is a significant limitation, as reliance on a single fixed 70/30 split may introduce bias in model performance evaluation. Moreover, no formal statistical tests were conducted to determine whether differences among models are statistically significant. The presence of identical MSE and R² values for both training and test sets in certain cases requires methodological clarification, as this may indicate overfitting, data leakage, or inconsistencies in model implementation.

Concerning data availability, although the manuscript states that the data are provided within the article, there is no explicit disclosure of the raw data underlying the reported results. The individual hardness values for the 81 specimens are not fully presented, nor is a complete dataset (including input variables and corresponding outputs) provided as a supplementary file. Additionally, the specific training and testing datasets used for the machine learning models are not made available, and no public repository with DOI or access link is indicated. Therefore, the data cannot be considered fully accessible in compliance with the PLOS Data Policy.

Furthermore, while biomedical applications are referenced, they are discussed in a conceptual manner without functional validation, clinical contextualization, or correlation with relevant biomedical standards or regulatory requirements. This weakens the practical significance claimed in the manuscript.

From a language perspective, although the manuscript is generally intelligible, it contains recurring linguistic issues that compromise the clarity and precision expected in international scientific publications. These include grammatical errors, awkward sentence constructions, overly long and complex sentences, inconsistent terminology (e.g., “stall orientation” instead of “raster orientation”), and minor descriptive inconsistencies in the results section. Given that PLOS ONE does not perform copyediting after acceptance, the manuscript requires professional native-level English editing prior to publication.

In summary, the study demonstrates technical merit and a reasonably organized methodological framework suitable for an exploratory investigation. However, it requires stronger statistical rigor, improved experimental validation, enhanced data transparency, and substantial linguistic refinement in order to meet the standards expected of higher-impact scientific journals.

From a rigorous scientific and methodological standpoint, the manuscript should not be accepted in its current form, but it also does not warrant outright rejection. The study presents a structured experimental design and a technically valid comparative machine learning framework, which indicates underlying scientific merit. However, several substantive weaknesses prevent immediate acceptance.

Reviewer #2: 01 - The information on PMMA presented at the beginning of the methodology could be reorganized into a table format, which would contribute to greater clarity and improve the overall flow of the text.

02 - Although the authors indicate the variables that were modified and investigated, it is not clear how these variables are combined in each experimental condition. For example, when varying the layer height, the values adopted for parameters such as infill pattern and printing orientation are not specified. These are relevant details that should be explicitly stated to ensure the reproducibility of the study.

03 - Additionally, no information is provided regarding the use of sample replicates or the number of indentations performed per sample. The absence of these details compromises the assessment of the statistical rigor of the study.

Considering that the CatBoost model showed superior performance after optimization, what factors could explain its advantage over XGBoost when techniques such as Optuna and skOpt are applied? It would be beneficial to elaborate further on this aspect in the manuscript.

**********

what does this mean?). If published, this will include your full peer review and any attached files.

If you choose “no”, your identity will remain anonymous but your review may still be made public.

**Do you want your identity to be public for this peer review?**  For information about this choice, including consent withdrawal, please see our Privacy Policy

Reviewer #1: **Yes:**  Romulo Maziero

Reviewer #2: No

**********

[NOTE: If reviewer comments were submitted as an attachment file, they will be attached to this email and accessible via the submission site. Please log into your account, locate the manuscript record, and check for the action link "View Attachments". If this link does not appear, there are no attachment files.]

To ensure your figures meet our technical requirements, please review our figure guidelines: https://journals.plos.org/plosone/s/figures

You may also use PLOS’s free figure tool, NAAS, to help you prepare publication quality figures: https://journals.plos.org/plosone/s/figures#loc-tools-for-figure-preparation.

NAAS will assess whether your figures meet our technical requirements by comparing each figure against our figure specifications.

# Author comment: Author response to Decision Letter 1

**DOI:** 10.1371/journal.pone.0355862.r002

6 May 2026

Reviewer #1

1. The manuscript presents a methodologically structured proposal, with a clearly defined experimental design involving 81 specimens fabricated under systematic variation of printing parameters (layer height, infill density, infill pattern, and raster orientation), as well as a 70/30 train–test data split. The comparison of multiple machine learning models (Linear Regression, Decision Tree, SVR, AdaBoost, XGBoost, and CatBoost), including hyperparameter optimization using Optuna and skOpt, represents a positive aspect of the study. The evaluation metrics employed (MSE and R²), along with residual analysis, are appropriate for assessing predictive performance. Furthermore, the overall organization of the manuscript (Introduction, Methodology, Results, and Conclusion) follows a logical scientific structure. However, although the data support the conclusions within the experimental scope presented, the study exhibits methodological and statistical limitations that prevent it from being considered fully robust under the rigorous standards required for high-impact international publication. From an experimental standpoint, there is no detailed description of replicate hardness measurements, nor is there any reporting of experimental variability (e.g., standard deviation, standard error, or repeatability analysis). Additionally, no formal inferential statistical analyses were conducted, such as ANOVA, significance testing, or confidence interval estimation, which would allow a more rigorous evaluation of the effects of printing parameters and their potential interactions. The sample size (81 specimens) is relatively limited for robust generalization in machine learning applications, particularly given the complexity of the algorithms employed.

Response 1:

Thank you for the comment. In this experiment, the hardness values were recorded thrice on different spots of the specimen. This was made to ensure that any potential error due to local variations is taken into account. The values of hardness used in the analysis are the average of these three measurements. Moreover, a full factorial combination table has been included in Annexure 1 with the average value of hardness. Besides improving precision, a descriptive statistical analysis has also been done by including the mean and standard deviation. Although this research is based on predictions using machine learning, an analysis of the parameters' impact through statistics has also been included briefly.

Manuscript changes in Manuscript (Methodology Section)

In this research work, hardness values for the corresponding specimens were recorded thrice on different spots. This was made to ensure that any potential error due to local variations is taken into account. The values of hardness used in the analysis are the average of these three measurements. Moreover, a full factorial combination table has been included in Annexure 1 with the average value of hardness. Besides improving precision, a descriptive statistical analysis has also been done by including the mean and standard deviation. Although this research is based on predictions using machine learning models, an analysis of the parameters' impact through statistics has also been considered.

2. Regarding the statistical modeling, although appropriate performance metrics and hyperparameter optimization procedures were used, the analysis cannot be considered fully rigorous. The absence of cross-validation (e.g., k-fold cross-validation) is a significant limitation, as reliance on a single fixed 70/30 split may introduce bias in model performance evaluation. Moreover, no formal statistical tests were conducted to determine whether differences among models are statistically significant. The presence of identical MSE and R² values for both training and test sets in certain cases requires methodological clarification, as this may indicate overfitting, data leakage, or inconsistencies in model implementation.

Response 2:

We agree that cross-validation is widely utilized for model validation. Nevertheless, in this specific instance, it should be noted that the data set was formed as a result of a properly designed full factorial experiment, which means that each possible combination of parameters occurs once and is included in the DOE. With regard to such data sets, the use of random split into folds in k-fold cross-validation might disturb the balance between combinations and yield subsets without complete representation of the design space.

With regards to the formal statistical tests, we concur that the inferential statistics can provide additional insight into the statistical significance of any differences between models under evaluation. It should, however, be noted that the purpose of our analysis was specifically to investigate the prediction abilities of various machine learning models in estimating hardness of a product using process parameters, rather than comparing the statistical significance of their results. The comparison of ML models involved widely used indicators of prediction such as MSE and R², as well as residual analysis. Moreover, a similar trend observed with multiple ML models and optimization methods (Optuna, SkOpt) could serve as an indicator of robustness.

Furthermore, it should be observed that the data set has been obtained through a well-designed full factorial experiment, which highlights the importance of the relationship between the process and its properties more than anything else. Due to the small sample size, inferential statistics may not prove to be useful or appropriate at all.

Manuscript changes in Methodology (ML Section)

Though the method of k-fold cross-validation is widely used during the generalization of machine learning models, in this research, the approach was voluntarily avoided. This was due to the nature of the experimental data obtained. A full-factorial design of experiments (DOE) was followed in the current research. All process parameter combinations were considered in the experimental DOE dataset. Random partitioning used in k-fold cross-validation might violate the balance between datasets and produce sets where some combinations are not sufficiently represented. Thus, to ensure the generalization capability, a controlled train–test split in a ratio of 70:30 was employed, where all levels and parameter combinations, including layer height, infill density, infill pattern, and raster orientation, were considered in both parts. The model assessment was conducted based on the metrics of MSE and R², complemented with residual analysis to make sure there is no bias. Similarly, MSE and R² values observed in this case for both training and testing data were related to the deterministic nature of some models and balanced uniform data distribution.

3. Concerning data availability, although the manuscript states that the data are provided within the article, there is no explicit disclosure of the raw data underlying the reported results. The individual hardness values for the 81 specimens are not fully presented, nor is a complete dataset (including input variables and corresponding outputs) provided as a supplementary file. Additionally, the specific training and testing datasets used for the machine learning models are not made available, and no public repository with DOI or access link is indicated. Therefore, the data cannot be considered fully accessible in compliance with the PLOS Data Policy

Response 3:

Thank you for noting the point. The complete dataset, including input parameters and corresponding hardness values, has now been provided in Annexure 1 to ensure transparency and reproducibility.

Manuscript changes:

The complete dataset used in this study, including input process parameters and corresponding hardness values, has been provided in Annexure 1.

4. Furthermore, while biomedical applications are referenced, they are discussed in a conceptual manner without functional validation, clinical contextualization, or correlation with relevant biomedical standards or regulatory requirements. This weakens the practical significance claimed in the manuscript

Response 4:

Thank you for making this useful comment. To solve the above-mentioned issue, there have been necessary changes made to the manuscript. The detailed explanation of the relationship between the estimated mechanical property of hardness and its relevance from a biomedical point of view is included. As far as applications of biomedical implants, including dentistry, artificial bones, etc., are concerned, hardness plays a key role in determining their wear resistance and strength. These details have been included in the manuscript now.

Manuscript changes in Introduction

The property of hardness can be very practical in relation to the field of biomedical engineering. This is especially true regarding the usage of materials such as PMMA in manufacturing dental prosthesis, bone replacement structures, and even customized biomedical parts. When considering the biomedical application, hardness becomes an extremely important attribute in determining wear resistance when working with any parts that will experience constant rubbing or friction against something else, such as dental crowns or dentures. Hardness is another factor in ensuring that the surface is durable and resistant to deformation. Regarding bone replacement structures and implants, having a certain degree of hardness ensures their compatibility. Thus, predicting hardness according to the parameters of manufacture makes it possible to optimize parts manufactured by additive manufacturing processes.

5. From a language perspective, although the manuscript is generally intelligible, it contains recurring linguistic issues that compromise the clarity and precision expected in international scientific publications. These include grammatical errors, awkward sentence constructions, overly long and complex sentences, inconsistent terminology (e.g., “stall orientation” instead of “raster orientation”), and minor descriptive inconsistencies in the results section. Given that PLOS ONE does not perform copyediting after acceptance, the manuscript requires professional native-level English editing prior to publication.

Response 5:

Thank you for the feedback. The manuscript has been thoroughly revised for grammar, clarity, and terminology consistency.

6. In summary, the study demonstrates technical merit and a reasonably organized methodological framework suitable for an exploratory investigation. However, it requires stronger statistical rigor, improved experimental validation, enhanced data transparency, and substantial linguistic refinement in order to meet the standards expected of higher-impact scientific journals

Response 6:

Thank you for the complete assessment of the work. In response to the concerns raised, the manuscript has been completely revised and strengthened in several key aspects.

Further clarifications on the statistical methods employed have been added to make them more rigorous. In particular, three hardness tests were performed per sample, and the numbers presented herein refer to the mean value with the standard deviation as a measure of variability. Also, a model evaluation has been performed by analyzing residuals and comparing results obtained using different machine learning approaches and optimization methods. Finally, the limitations relating to the lack of inferential statistics testing and the use of cross-validation have been discussed.

On the other hand, in relation to experimental validation, a description of the methodology applied, including the testing strategy and parameters involved, has been included. The full factorial design guarantees thorough sampling of the process domain.

To provide data transparency, the whole set of collected data containing all the input parameters as well as their respective hardness levels were included in Annexure 1.

Also, numerous linguistic changes have been made throughout the text in order to eliminate grammatical errors and improve readability.

We believe that the above changes have considerably increased the quality of the manuscript, and it is ready for submission.

7. From a rigorous scientific and methodological standpoint, the manuscript should not be accepted in its current form, but it also does not warrant outright rejection. The study presents a structured experimental design and a technically valid comparative machine learning framework, which indicates underlying scientific merit. However, several substantive weaknesses prevent immediate acceptance.

Response 7:

We thank you for your thorough assessment of the manuscript. Based on your concerns about the flaws found, we have made substantial changes to our manuscript.

Improvements have been made through better explanations of experimental replications and adding variability values, such as the average and standard deviation. Our methods have been elaborated upon to increase clarity and reproducibility. We have also ensured data transparency by including our full dataset in Annexure 1. Additionally, substantial corrections have been made in language for clarity purposes. We hope that these improvements have addressed all of your concerns and will be able to publish our work.

Reviewer #2:

1. The information on PMMA presented at the beginning of the methodology could be reorganized into a table format, which would contribute to greater clarity and improve the overall flow of the text.

Response 1

Thank you for the suggestion provided. The material properties are now presented in tabular form and are included in the manuscript under the methodology section

Manuscript changes in Methodology

Property Value

Density 1.18 g/cm³

Thermal Conductivity 0.19 W/mK

Specific Heat 1.47 J/gK

Print Temp 240–260°C

Bed Temp 80–100°C

2. Although the authors indicate the variables that were modified and investigated, it is not clear how these variables are combined in each experimental condition. For example, when varying the layer height, the values adopted for parameters such as infill pattern and printing orientation are not specified. These are relevant details that should be explicitly stated to ensure the reproducibility of the study.

Response 2:

Thank you for the useful comment. A full factorial combination table has now been added.

Manuscript changes

The complete dataset used in this study, including input process parameters and corresponding hardness values, has been provided in Annexure 1.

3. Additionally, no information is provided regarding the use of sample replicates or the number of indentations performed per sample. The absence of these details compromises the assessment of the statistical rigor of the study. Considering that the CatBoost model showed superior performance after optimization, what factors could explain its advantage over XGBoost when techniques such as Optuna and skOpt are applied? It would be beneficial to elaborate further on this aspect in the manuscript

Response 3:

Thank you for your comments. For the purpose of this experiment, data regarding the hardness of the test material have been collected from three different points. This was to account for any error due to local variations in the process. All the values of the hardness used for calculation were based on the average of these three points. An entire factorial design table, along with the average hardness values, has been included in Annexure 1 as well. There has not only been an improvement in terms of precision, but the use of statistics in terms of average and standard deviation has allowed for a descriptive analysis of the problem. Despite the nature of this study as one involving prediction through Machine Learning, a statistical analysis of parameters has also been provided.

In terms of the performance of ML models, CatBoost is designed in such a way that it handles categorical data in a more efficient manner. Since the use of parameters like infill pattern involves categorical data, CatBoost is better suited for this study than XGBoost..

CatBoost uses ordered boosting that prevents any prediction shift and minimizes overfitting, particularly helpful in the case of small and well-structured datasets like the f

Attachment Submitted filename: Responses to Reviewers.docx

# Peer review: Decision Letter 1

**DOI:** 10.1371/journal.pone.0355862.r003

**Authors:** André Gustavo Galdino

20 May 2026

Dear Dr. Katiyar,

Thank you for submitting your manuscript to PLOS ONE. After careful consideration, we feel that it has merit but does not fully meet PLOS ONE’s publication criteria as it currently stands. Therefore, we invite you to submit a revised version of the manuscript that addresses the points raised during the review process.

Please submit your revised manuscript by Jul 04 2026 11:59PM. If you will need more time than this to complete your revisions, please reply to this message or contact the journal office at plosone@plos.org. When you're ready to submit your revision, log on to https://www.editorialmanager.com/pone/ and select the 'Submissions Needing Revision' folder to locate your manuscript file.

- A letter that responds to each point raised by the academic editor and reviewer(s). You should upload this letter as a separate file labeled 'Response to Reviewers'.
- A marked-up copy of your manuscript that highlights changes made to the original version. You should upload this as a separate file labeled 'Revised Manuscript with Track Changes'.
- An unmarked version of your revised paper without tracked changes. You should upload this as a separate file labeled 'Manuscript'.

If you would like to make changes to your financial disclosure, please include your updated statement in your cover letter. Guidelines for resubmitting your figure files are available below the reviewer comments at the end of this letter.

If applicable, we recommend that you deposit your laboratory protocols in protocols.io to enhance the reproducibility of your results. Protocols.io assigns your protocol its own identifier (DOI) so that it can be cited independently in the future. For instructions see: https://journals.plos.org/plosone/s/submission-guidelines#loc-laboratory-protocols. Additionally, PLOS ONE offers an option for publishing peer-reviewed Lab Protocol articles, which describe protocols hosted on protocols.io. Read more information on sharing protocols at https://plos.org/protocols?utm_medium=editorial-email&utm_source=authorletters&utm_campaign=protocols.

As the corresponding author, your ORCID iD is verified in the submission system and will appear in the published article. PLOS supports the use of ORCID, and we encourage all coauthors to register for an ORCID iD and use it as well. Please encourage your coauthors to verify their ORCID iD within the submission system before final acceptance, as unverified ORCID iDs will not appear in the published article. *Only*  the individual author can complete the verification step; PLOS staff *cannot*  verify ORCID iDs on behalf of authors.

We look forward to receiving your revised manuscript.

Kind regards,

André Gustavo de Sousa Galdino

Academic Editor

PLOS One

Journal Requirements:

If the reviewer comments include a recommendation to cite specific previously published works, please review and evaluate these publications to determine whether they are relevant and should be cited. There is no requirement to cite these works unless the editor has indicated otherwise.

**Additional Editor Comments:**

Please read reviewers' comments and improve your manuscript.

[Note: HTML markup is below. Please do not edit.]

Reviewers' comments:

Reviewer's Responses to Questions

**Comments to the Author**

Reviewer #1: (No Response)

Reviewer #2: All comments have been addressed

**********

2. Is the manuscript technically sound, and do the data support the conclusions?

Reviewer #1: Partly

Reviewer #2: Yes

**********

3. Has the statistical analysis been performed appropriately and rigorously?

Reviewer #1: No

Reviewer #2: Yes

**********

4. Have the authors made all data underlying the findings in their manuscript fully available?

The PLOS Data policy

Reviewer #1: Yes

Reviewer #2: Yes

**********

5. Is the manuscript presented in an intelligible fashion and written in standard English?

Reviewer #1: Yes

Reviewer #2: Yes

**********

Reviewer #1: Overall, the authors addressed most of the reviewers’ comments and implemented several meaningful improvements to the manuscript, particularly regarding: inclusion of experimental replicates; addition of mean and standard deviation values; clarification of the CatBoost model performance; inclusion of the complete dataset in the appendix; improvement of language and manuscript organization; expanded biomedical contextualization of PMMA hardness. Nevertheless, several responses remain only partially satisfactory from a scientific standpoint.

The authors did not perform: ANOVA; statistical significance testing; confidence interval analysis; formal interaction effect analysis. Instead, they mainly justified the absence of these methods. From a scientific perspective, this considerably weakens the robustness of the experimental interpretation. For a full-factorial DOE, the absence of ANOVA is particularly problematic, since this type of experimental design is specifically intended for evaluating parameter effects and interactions.

The justification provided for not using k-fold cross-validation is not entirely convincing. For small datasets, cross-validation is generally recommended precisely to reduce train-test split bias. Moreover, several alternative approaches could have been considered, including: stratified validation; repeated k-fold validation; leave-one-out validation; grouped cross-validation. Although the explanation may be acceptable during revision, it remains methodologically weak.

Several statistically questionable results are still present. For example: multiple models exhibit exactly identical training and testing metrics; some cases report R² = 1.0000; XGBoost optimization reports MSE = 0.0000. Such results are highly unusual for real experimental datasets. Even though the authors attempted to justify these findings based on balanced data distribution, there are still strong indications of: overfitting; data leakage; or excessive dataset simplification. This issue would still require deeper methodological clarification.

Immediate acceptance is not recommended. The manuscript still requires moderate methodological revision.

Reviewer #2: All questions were answered satisfactorily. Therefore, I believe the article will be a good contribution to the scientific community and I recommend its publication.

**********

what does this mean?). If published, this will include your full peer review and any attached files.

If you choose “no”, your identity will remain anonymous but your review may still be made public.

**Do you want your identity to be public for this peer review?**  For information about this choice, including consent withdrawal, please see our Privacy Policy

Reviewer #1: No

Reviewer #2: No

**********

[NOTE: If reviewer comments were submitted as an attachment file, they will be attached to this email and accessible via the submission site. Please log into your account, locate the manuscript record, and check for the action link "View Attachments". If this link does not appear, there are no attachment files.]

To ensure your figures meet our technical requirements, please review our figure guidelines: https://journals.plos.org/plosone/s/figures

You may also use PLOS’s free figure tool, NAAS, to help you prepare publication quality figures: https://journals.plos.org/plosone/s/figures#loc-tools-for-figure-preparation.

NAAS will assess whether your figures meet our technical requirements by comparing each figure against our figure specifications.

# Author comment: Author response to Decision Letter 2

**DOI:** 10.1371/journal.pone.0355862.r004

4 Jul 2026

REVIEWER #1:

COMMENT 1:

The authors did not perform: ANOVA; statistical significance testing; confidence interval analysis; formal interaction effect analysis. Instead, they mainly justified the absence of these methods. From a scientific perspective, this considerably weakens the robustness of the experimental interpretation. For a full-factorial DOE, the absence of ANOVA is particularly problematic, since this type of experimental design is specifically intended for evaluating parameter effects and interactions.

RESPONSE 1:

We thank the reviewer for providing the valuable suggestion. A complete statistical analysis has been carried out in the revised manuscript.

The following analyses have been reported:

● ANOVA test with full factorial design for testing the statistical significance of all four printing parameters.

● ANOVA test with Interaction design for testing the statistical significance of interaction between the process parameters.

● 95% Confidence Interval Analysis for the hardness data.

● Interaction Effect Analysis along with Interaction plots.

● Statistical significance was tested at 95% confidence level.

The results obtained from ANOVA tests show that there is a significant effect of layer height, infill density, shell thickness, and print orientation on the hardness. Several interaction terms are also statistically significant.

The ANOVA tables, interaction effect analysis, confidence interval analysis, and discussions are included in the revised manuscript as a separate section entitled “ANOVA Analysis”

Thereby, the statistical analysis of experimental results has been significantly improved.

COMMENT 2:

The justification provided for not using k-fold cross-validation is not entirely convincing. For small datasets, cross-validation is generally recommended precisely to reduce train-test split bias. Moreover, several alternative approaches could have been considered, including: stratified validation; repeated k-fold validation; leave-one-out validation; grouped cross-validation. Although the explanation may be acceptable during revision, it remains methodologically weak.

RESPONSE 2:

We thank the reviewer for this important observation.

On the basis of this comment, the machine learning algorithm has been revised using 10-fold cross-validation for each machine learning model examined.

The new manuscript now contains the following information:

● 10-Fold Cross Validation approach

● Cross Validation MSE

● Cross Validation R²

● Comparison of the initial train-test method and 10-fold cross validation

● Generalization capability discussion

The cross-validation has shown that the optimized models keep their high predictive capabilities even when applied to new data. The close coincidence of the performance in the test method and in the cross-validation proves that the developed models have good generalization capability.

A new section "Comparative Performance of Models after cross validation" has been introduced into the manuscript.

COMMENT 3:

Several statistically questionable results are still present. For example: multiple models exhibit exactly identical training and testing metrics; some cases report R² = 1.0000; XGBoost optimization reports MSE = 0.0000. Such results are highly unusual for real experimental datasets. Even though the authors attempted to justify these findings based on balanced data distribution, there are still strong indications of: overfitting; data leakage; or excessive dataset simplification. This issue would still require deeper methodological clarification.

RESPONSE 3:

We appreciate the reviewer's careful examination of the reported machine learning results and bringing it into our notice.

In response to this comment, we critically analyzed all aspects of the machine learning pipeline, from data preparation to model building, optimization, and validation. A number of key points have been included into the revised version of the manuscript. The presented R² ≈ 1.0000 and MSE ≈ 0.0000 values refer to training-set results only for highly adaptive ensemble methods following Bayesian hyperparameter optimization procedure. This data was not used to evaluate the predictive ability of the models. In order to check whether the achieved results could be the consequence of overfitting or data leakage, we used 10-fold cross-validation procedure for all of the developed models. The outcomes of the cross-validation prove that the optimized models provide high performance on unseen data.

The differences between testing performance and cross-validation performance are small, indicating excellent model robustness and limited evidence of severe overfitting. Additionally, the complete preprocessing pipeline was re-examined to eliminate potential data leakage. Hyperparameter optimization and model fitting were performed exclusively using the training data within each validation fold, while the validation fold remained completely unseen during training. Thus the discussion has been revised to emphasize testing performance and cross-validation performance rather than training metrics, since these provide a more realistic assessment of model generalization. Accordingly, we believe that the additional validation significantly strengthens the statistical credibility of the proposed machine learning framework. This explanation has been added in the manuscript as a separate section entitled “Model Generalization and Overfitting”

Attachment Submitted filename: Response to Reviewer.docx

# Peer review: Decision Letter 2

**DOI:** 10.1371/journal.pone.0355862.r005

**Authors:** André Gustavo Galdino

27 Jul 2026

Machine Learning Models for Predicting the Hardness of Additively Manufactured Polymethyl Methacrylate (PMMA) Parts for Biomedical Applications

PONE-D-26-07638R2

Dear Dr. Katiyar,

We’re pleased to inform you that your manuscript has been judged scientifically suitable for publication and will be formally accepted for publication once it meets all outstanding technical requirements.

Within one week, you’ll receive an e-mail detailing the required amendments. When these have been addressed, you’ll receive a formal acceptance letter and your manuscript will be scheduled for publication.

An invoice will be generated when your article is formally accepted. Please note, if your institution has a publishing partnership with PLOS and your article meets the relevant criteria, all or part of your publication costs will be covered. Please make sure your user information is up-to-date by logging into Editorial Manager at Editorial Manager® and clicking the ‘Update My Information' link at the top of the page. For questions related to billing, please contact billing support.

If your institution or institutions have a press office, please notify them about your upcoming paper to help maximize its impact. If they’ll be preparing press materials, please inform our press team as soon as possible -- no later than 48 hours after receiving the formal acceptance. Your manuscript will remain under strict press embargo until 2 pm Eastern Time on the date of publication. For more information, please contact onepress@plos.org.

Kind regards,

André Gustavo de Sousa Galdino

Academic Editor

PLOS One

Additional Editor Comments (optional):

Reviewers' comments:

Reviewer's Responses to Questions

**Comments to the Author**

Reviewer #1: All comments have been addressed

Reviewer #2: All comments have been addressed

**********

2. Is the manuscript technically sound, and do the data support the conclusions?

Reviewer #1: Yes

Reviewer #2: Yes

**********

3. Has the statistical analysis been performed appropriately and rigorously?

Reviewer #1: Yes

Reviewer #2: Yes

**********

4. Have the authors made all data underlying the findings in their manuscript fully available?

The PLOS Data policy

Reviewer #1: Yes

Reviewer #2: Yes

**********

5. Is the manuscript presented in an intelligible fashion and written in standard English?

Reviewer #1: Yes

Reviewer #2: Yes

**********

Reviewer #1: I appreciate the authors' considerable efforts in revising the manuscript. The revised version adequately addresses the major concerns raised during the previous review round. In particular, the inclusion of a comprehensive ANOVA analysis, interaction effect analysis, confidence interval evaluation, and 10-fold cross-validation substantially improves the methodological rigor of the study. The additional discussion regarding model generalization, overfitting, and data leakage also strengthens the reliability of the machine learning results.

The manuscript is technically sound, the experimental methodology is appropriate, and the conclusions are supported by the presented data. The comparison of different machine learning models, together with hyperparameter optimization and statistical validation, provides a valuable contribution to the prediction of hardness in additively manufactured PMMA components for biomedical applications.

Although a few minor language and stylistic improvements could still be made during the production stage, these do not affect the scientific quality of the work. Overall, I believe the manuscript now meets the standards required for publication in PLOS ONE, and I have no further technical comments.

Reviewer #2: (No Response)

**********

what does this mean?). If published, this will include your full peer review and any attached files.

If you choose “no”, your identity will remain anonymous but your review may still be made public.

**Do you want your identity to be public for this peer review?**  For information about this choice, including consent withdrawal, please see our Privacy Policy

Reviewer #1: No

Reviewer #2: No

**********

# Editor report: Acceptance letter

**DOI:** 10.1371/journal.pone.0355862.r006

**Authors:** André Gustavo Galdino

PONE-D-26-07638R2

PLOS One

Dear Dr. Katiyar,

I'm pleased to inform you that your manuscript has been deemed suitable for publication in PLOS One. Congratulations! Your manuscript is now being handed over to our production team.

At this stage, our production department will prepare your paper for publication. This includes ensuring the following:

* All references, tables, and figures are properly cited

* All relevant supporting information is included in the manuscript submission,

* There are no issues that prevent the paper from being properly typeset

You will receive further instructions from the production team, including instructions on how to review your proof when it is ready. Please keep in mind that we are working through a large volume of accepted articles, so please give us a few days to review your paper and let you know the next and final steps.

Lastly, if your institution or institutions have a press office, please let them know about your upcoming paper now to help maximize its impact. If they'll be preparing press materials, please inform our press team within the next 48 hours. Your manuscript will remain under strict press embargo until 2 pm Eastern Time on the date of publication. For more information, please contact onepress@plos.org.

You will receive an invoice from PLOS for your publication fee after your manuscript has reached the completed accept phase. If you receive an email requesting payment before acceptance or for any other service, this may be a phishing scheme. Learn how to identify phishing emails and protect your accounts at https://explore.plos.org/phishing.

If we can help with anything else, please email us at customercare@plos.org.

Thank you for submitting your work to PLOS ONE and supporting open access.

Kind regards,

PLOS ONE Editorial Office Staff

on behalf of

Dr. André Gustavo de Sousa Galdino

Academic Editor

PLOS One
